# An Algorithm for Emulsion Stability Simulations: Account of Flocculation, Coalescence, Surfactant Adsorption and the Process of Ostwald Ripening

**DOI:** 10.3390/ijms10030761

**Published:** 2009-02-26

**Authors:** German Urbina-Villalba

**Affiliations:** Instituto Venezolano de Investigaciones Científicas (IVIC), Centro de Física, Lab. Fisicoquímica de Coloides, Aptdo. 20632, Caracas, Venezuela; E-Mail: guv@ivic.ve; Tel. +58-212-504-1542; Fax: +58-212-504-1148

**Keywords:** Flocculation, Coalescence, Adsorption, Surfactant, Drops, Ostwald, Simulations, Emulsion, Deformation

## Abstract

The first algorithm for Emulsion Stability Simulations (ESS) was presented at the V Conferencia Iberoamericana sobre Equilibrio de Fases y Diseño de Procesos [Luis, J.; García-Sucre, M.; Urbina-Villalba, G. Brownian Dynamics Simulation of Emulsion Stability In: *Equifase 99. Libro de Actas*, 1^st^ Ed., Tojo J., Arce, A., Eds.; Solucion’s: Vigo, Spain, 1999; Volume 2, pp. 364–369]. The former version of the program consisted on a minor modification of the Brownian Dynamics algorithm to account for the coalescence of drops. The present version of the program contains elaborate routines for time-dependent surfactant adsorption, average diffusion constants, and Ostwald ripening.

## 1. Introduction

Emulsions are dispersions of two immiscible liquids, kinetically stabilized by the action of a surface-active substance known as a surfactant. The role of the surfactant in emulsion stability is decisive. Even the external phase of the emulsion resulting from the mixing of oil (O) and water (W) depends on the surfactant solubility [1–5]. In the absence of surfactants, drops aggregate rapidly as a consequence of the van der Waals force. Surfactants adsorb to the interface of the drops creating a repulsive barrier that decelerate aggregation and Ostwald ripening. They also favor the occurrence of immobile interfaces, delaying the drainage of the intervening film between flocculated drops [6]. Conversely, surfactants lower the interfacial tension of these O/W/O films, favoring the appearance of surface oscillations and holes. Depending on their interfacial properties, these films can drain and rupture or remain stable for long periods of time [7–10].

In order to understand the role of the surfactant in the stability of emulsions, simulations can be a valuable tool. However, there are severe computational restrictions for the simulation of emulsions apart from the large number of processes involved. First, the number of surfactant molecules in a typical system is very high even at dilute concentrations. Hence, it is not possible to simulate the movement of surfactant molecules explicitly along with the movement of the drops. Second, the time step of the simulation has to be very small in order to sample appropriately the potential of interaction between the drops. Third, drops of small size exhibit Brownian motion due to their thermal interaction with the solvent [11]. This has to be taken into account into their equation of motion. Fourth, it is not possible to simulate the behavior of drops using a constant potential as it is done in molecular dynamics. The potential of interaction changes with time as a consequence of surfactant adsorption and the progressive decrease of interfacial area of the emulsion due to the coalescence of drops. This demands the frequent calculation of the interfacial properties that determine the forces between the particles.

In this review we concentrate on the evolution of oil-in-water (O/W) emulsions composed of non-deformable drops. The next section introduces the technique of Brownian Dynamics simulations and the most relevant aspects of the classical theory of Derjaguin-Landau-Verwey-Overbeek (DLVO). Section 3 describes the algorithm of Emulsion Stability Simulations (ESS) in detail. In section 4 some illustrative results of ESS are discussed. Following, the modifications of the former algorithm required for the simulation of deformable drops are outlined. The paper finishes with a brief conclusion and the bibliography.

## 2. Brownian Dynamics (BD) Simulations

The movement of one small particle (1 nm – 10 μm) diffusing in a quiescent media is the result of external and random forces. Random forces represent the effect of millions of collisions occurring between the solvent molecules and the particle surface. In the absence of external forces the energy for the displacement of a spherical particle is provided by the solvent, which at the same time takes away some energy from the particle in the form of friction. The whole process is a manifestation of the Fluctuation-Dissipation theorem that is expressed concisely in the form of the Stokes-Einstein equation [11–12]:

(1) $$\tilde{D}\;f\;\;=\;k_{B}\;T$$

In this equation, *D͂* and *f* stand for the diffusion tensor and the resistance tensor of the particle (or a set of particles), and *k_B_* is the Boltzmann constant. In the case of a spherical particle the diffusion tensor is diagonal and its three components are equal (*D*). According to Equation (1), the absolute temperature of the reservoir (*T*) is kept constant while the movement of the particle occurs, although there is a continuous exchange of energy between the particle and the solvent. This stochastic process can be described analytically in terms of the Langevin equation [13]. The solution of Langevin’s equation for one spherical particle provides explicit expressions for its mean square displacement and its diffusion constant (*D*). The diffusion constant turns out to be proportional to the temporal correlation of the random fluctuations of the particle movement. The overall effect of the interaction between a suspended particle and the surrounding molecules is a random drift in the trajectory of the particle, whose displacement shows a Gaussian distribution with zero mean and a standard deviation equal to $\sqrt{2D\Delta t}$ along each co-ordinate axis. Here Δ*t* is the lapse of time considered and *D* is the diffusion constant of a solitary particle [13].

The diffusion tensor can be evaluated from the resistance tensor: *D͂* = *f*^−1^*k_B_* *T* for a particle moving through the liquid as a consequence of external, hydrodynamic or interparticle forces. In turn, the resistance tensor can be obtained from the drag force experienced by the particle when it moves through the fluid at a constant velocity v [14]:

(2) F  = −f v

The specific form of the diffusion constant of a sphere depends on the velocity of the liquid at the particle surface. If the surface is rigid and smooth, the velocity of the fluid becomes zero at the surface. In this case the diffusion constant comes out to *be D* = *D*_0_ =*k_B_* *T/6πη R,* where: *η* and *R* are the shear viscosity of the solvent and the radius of the particle, respectively.

In the case of liquid drops, porous spheres, and bitumen drops, the diffusion constant can be expressed as:

(3) $$D=D_{0}\;f_{corr}^{\left(1\right)}$$

where $f_{\text{corr}}^{\left(1\right)}$ is a correction function depending on the characteristics of the O/W interface [14].

The addition of more particles to the system increases the complexity of the problem considerably. First, the random movement of the particles must be connected in such a way that they fulfil the Stokes-Einstein relation. Second, the movement of each particle generates fluxes (disturbances) in the solvent which affect the movement of the surrounding particles and its own. Thus, it is necessary to account for hydrodynamic interactions between the particles. Third, the particles interact with deterministic forces other than external forces. Thus, their movement is a combination of deterministic hydrodynamic and random forces.

One of the most widely used algorithms for Brownian Dynamics simulations is the one of Ermak and McCammon [15]. In this formalism the position of a particle at time *t*+ Δ*t, r_i_* (*t* + Δ*t*) is equal to:

(4) $$r_{i}(t+\text{Δ}t)\;=\;r_{i}(t)\;+\sum_{j}\partial \;D_{ij}^{0}/\partial r_{j}\;+\sum_{j}\left[D_{ij}^{0}F_{j}^{0}/k_{B}T\right]\text{Δ}t\;+{\tilde{R}}_{i}(\text{Δ}t)$$

where the superscript “0” indicates that the variable is evaluated at the beginning of the time step. Subscripts *i* and *j* run over the particle coordinates (1 ≤ *i, j* ≤ 3*N*). *F_j_* is the sum of interparticle and external forces acting on direction *j. D_ij_* are the components of the diffusion tensor. The gradient of the diffusion tensor (second term on the right hand side) can usually made equal to zero selecting a tensor that depends on the distances between the particles and not on the particles coordinates. The third term on the right hand side stand for deterministic contributions and the fourth term correspond to the random displacements. A random deviate has the form:

(5) $${\tilde{R}}_{i}(\text{Δ}t)\;=\;\sum_{j}^{i}\sigma_{ij}X_{j}$$

Here *X_j_* stands for a random variable sampled from a Gaussian deviate generator: 〈*X_i_*〉 = 0, 〈*X_i_* *X_j_*〉 = 2 *δ_ij_* Δ*t*, where *δ_ij_* is the Kronecker delta. The weighting factors are given by:

(6) $$\sigma_{ii}\;={\left(D_{ii}-\sum_{k=i}^{i-1}\sigma_{ik}^{2}\right)}^{1/2}$$

(7) $$\sigma_{ij}\;=\;{\left(D_{ij}-\sum_{k=i}^{j-1}\sigma_{ik}\sigma_{jk}\right)}^{1/2}/\sigma_{jj}$$

According to the Ermak and McCammon equation (4) is compatible with a Fokker Planck description of the problem in the phase space. In the past, our group carried out the implementation of the above-mentioned algorithm with different tensors including Oseen, Rotne-Prager, and Batchelor’s [16] finding several limitations of this technique for emulsion stability calculations. Equations (5)–(7) describe a particular method for taking the square root of the diffusion tensor ( $\sqrt{2\tilde{D}\Delta t}$). However, the methodology suggested (Cholesky decomposition [17]) only works with particles of equal radii, and dilute systems. Other methods found in the bibliography like the QR decomposition also fail in simulations of polydispersed concentrated systems. This failure is caused by the assumption of pair wise additive hydrodynamic interactions. These schemes overestimate the hydrodynamic fluxes generated between the one central particle and its neighbours. They do not take into consideration that the flux coming from a particle located in the second coordination layer is partially screened by the inner neighbours. Dickinson suggested that the assumption of pair wise hydrodynamic interactions might even lead to negative diffusion constants [18].

When an average diffusion constant is used instead of a tensor, its value is de-coupled from the random deviates. Using this approximation and *D* = *D*_0_ *,* we quantified the kinetic energy of micron-size particles produced by the random fluctuations of the Box-Muller algorithm [17]. The kinetic energy was computed from the time step of the simulation and the displacement of each particle in the absence of deterministic and external forces. It was observed that fluctuations as high as 10,000 *k_B_T* must be allowed in order to reproduce a mean square displacement equal to: 〈*r^2^*〉 = *6D* Δ*t.* Cut off thresholds in the value of the deviates corresponding to kinetic energies of 1000 *k_B_T and* 100 *k_B_T* fail to reproduce the correct value of 〈*r^2^*〉. It was evident that values of the displacement corresponding to the outskirts of the Gaussian distribution are necessary in order to simulate the Brownian movement of the particles correctly [19].

The above considerations are profoundly related to the outcome of ESS calculations and its discussion is not a mere technicality. The most famous theory of colloidal stability, Derjaguin-Landau-Verwey-Overbeek’s DLVO theory [20] is based on the problem of diffusive passage over a potential barrier.

**Figure 1.**
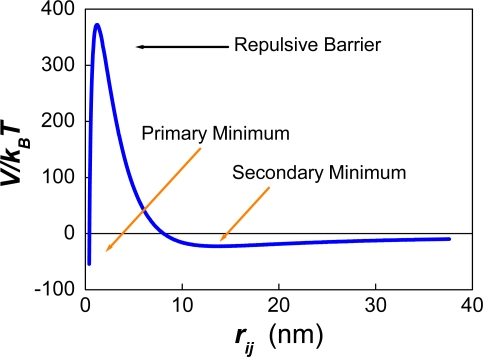
Classical DLVO potential. The curve corresponds to the interaction potential between two 3.9-μm drops of dodecane (*A_H_* = 5.03 × 10^−21^ J) suspended in water. The drops are partially covered with sodium dodecyl sulfate (*C_s_* = 10^−4^ M, *z_s_* = −0.2057) using a homogeneous surfactant distribution (Section 3).

The presence of a strong repulsive force between the particles generates a potential barrier and two minima at each side of the barrier (Figure 1).

On the one hand, primary minimum flocculation occurs at very small distances of separation and is assumed to be irreversible. Irreversible meaning that the aggregates formed do not separate if one lowers the ionic strength of the solution, increasing the repulsive force. This behaviour is due to the strong van der Waals force experienced by the particles at short distances of separation. On the other hand, secondary-minimum flocculation is usually reversible for small particles and could be “irreversible” for micron size drops [21]. Irreversible meaning in this case that the minimum is deep enough to prevent the particles from moving in and out of the potential well.

The occurrence of primary minimum flocculation depends on the diffusive passage of the particles over the potential barrier. According to Chandrasekhar [22] and Kramer [23] the probability of “jumping” over the barrier decreases exponentially with the size of the barrier. In order to account for the effect of the barrier on primary minimum flocculation, Fuchs [24] defined a “Stability ratio” *W*. The complete formula of *W* was deduced later by McGown and Parfitt [25]:

(8) $$W\;=\;k_{f}^{fast}/k_{f}^{slow}=\int_{2R_{i}}^{\infty }\left[f(r)/r^{2}\right]\text{exp}(V_{T}/k_{B}T)dr/\int_{2R_{i}}^{\infty }\left[f(r)/r^{2}\right]\text{exp}(V_{A}/k_{B}T)dr$$

Here *k_f_* stands for the flocculation rate. It can be either fast ( $k_{f}^{\text{fast}}$) or slow ( $k_{f}^{\text{slow}}$) if the potential of interaction between the particles is only due to attractive forces (*V_A_*) or caused by a combination of attractive and repulsive (*V_R_*) contributions (*V_T_* = *V_A_* + *V_R_*). In Equation (8) the dependence of the friction on the distance between the particles (*r*) has been remarked. Numerical evaluations of Equation (8) lead Prieve and Ruckenstein [26] to a very useful relation between the height of the repulsive barrier (*ΔV*) and the stability ratio:

(9) $$\text{log}(W)\;=\;0.40(\text{Δ}V/k_{B}T-1)$$

Fuchs demonstrated that the theory of Smoluchowski [27] for fast flocculation could be conveniently modified in order to incorporate the average effect of a repulsive barrier through the stability ratio. Thus, the change in the total number of aggregates of a dispersion per unit volume (*n*), can be written as:

(10) $$n(t)=\;n_{0}/\left(1+k_{f}^{fast}n_{0}t/W\right)$$

where *n_0_* = *n*(*t* = *0*).

According to Equations (8)–(10) a repulsive barrier produces large values of *W* which retard the attainment of primary minimum flocculation. A measure of colloid stability towards flocculation is given by the half lifetime of the dispersion *t_1/2_*. This is the time required for a decrease of *n_0_*/2 in the initial number of aggregates:

(11) $$t_{1/2}\;=\;W/k_{f}^{fast}n_{0}\;=\;1/k_{f}^{slow}n_{0}$$

In the case of non-deformable drops, primary minimum flocculation *implies coalescence*. Drops that jump over the repulsive barrier necessarily coalesce since there is no other repulsive force to prevent it. Due to the irreversible nature of the coalescence process the correct simulation of the diffusion tensor and the random deviates is critical.

## 3. Emulsion Stability Simulations (ESS)

### 3.1. Equation of Motion

Emulsion Stability Simulations start from a cubic box that contains *N* drops randomly distributed. The particles move with an equation of motion similar to the one of Brownian Dynamic Simulations [28–30]:

(12) $${\vec{r}}_{i}(t+\text{Δ}t)\;=\;{\vec{r}}_{i}(t)\;+\left\{\left(\sum_{j}{\vec{F}}_{ji}+{\vec{F}}_{ext,i}\right)D_{eff,i}(d,\varphi )/k_{B}T\right\}\text{Δ}t\;+\;\sqrt{2D_{eff,i}(d,\varphi )\text{Δ}t}\left[\vec{G}auss\right]$$

In Equation (12) subscripts *i* and *j* refer to particles *i* and *j.* The displacement of particle *i* during time $\Delta t:{\vec{r}}_{i}\left(t+\Delta t\right)\;\;-\;{\vec{r}}_{i}\left(t\right)$, is the result of two contributions. The second term on the right hand side of Equation (12) represents the effect of deterministic forces acting on particle *i*. It is composed of inter particle $\sum_{j}{\vec{F}}_{\text{ji}}$ and external forces ${\vec{F}}_{\text{ext},i}$. These forces move *i* with a constant velocity $\left(\sum_{j}{\vec{F}}_{\text{ji}}+{\vec{F}}_{\text{ext},i}\right)D_{\text{eff},i}\left(d,\varphi \right)/k_{B}$ *T* during time *Δt,* where *D_eff,i_* (*d, φ*) is an effective diffusion constant.

In the case of non-deformable particles *D_eff,i_*(*d, φ*) is calculated following the methodology described in Ref. [30]. At every step during the simulation the space around particle “*i* ” is divided in three spherical regions. If at least one neighbour particle reaches the internal region: *r_ij_* < *d* = *r*_int_ (where: $r_{ij}\;\;=\;\;\left|{\vec{r}}_{i}\left(t\right)\;\;-{\vec{r}}_{i}\left(t\right)\right|$), the formula of Honig et al. [31] is used to compute the diffusion constant of *i*. Otherwise the volume fraction of particles around *i* (*r*_int_ < *r_ij_* < *r_ext_*) is used to evaluate an empirical expression of *D_eff,i_*(*d, φ*) [30]. Particles located at *r_ij_* > *r_ext_* do not contribute to the hydrodynamic interactions of *i.* For spherical particles, *D_eff,i_* (*d, φ*) is equal to *D_eff,i_* (*d, φ*) = *D* $f_{\text{corr}}^{\left(2\right)}$, where *D* = *D*_0_ $f_{\text{corr}}^{\left(1\right)}$(Equation (3)). The first correction term ( $f_{\text{corr}}^{\left(1\right)}$) takes into account those factors that change the expression of the diffusion constant of a particle at infinite dilution (*D*) [14]. The second correction term $f_{\text{corr}}^{\left(2\right)}$ takes into account hydrodynamic interactions caused by the movement of the surrounding liquid as the particles move [30].

The program includes several forms for $f_{\text{corr}}^{\left(1\right)}$ and $f_{\text{corr}}^{\left(2\right)}$. However, in almost all simulations of non-deformable droplets previously reported, we assumed ideal spherical particles with zero tangential velocity at their surfaces, for which $f_{\text{corr}}^{\left(1\right)}$ = 1. Thus, we concentrated on the effect of the hydrodynamic interactions generated by the neighbour particles of *i, D_eff,i_* (*d,φ*) = *D*_0_ $f_{\text{corr}}^{\left(2\right)}$, with:

(13) $$f_{corr}^{\left(2\right)}=1.0-1.734\varphi +0.91\varphi^{2}\;\text{for}\;r_{\text{int}}\;<r_{ij}<r_{ext},$$

And:

(14) $$f_{corr}^{\left(2\right)}\;=\;\left(6u^{2}+4u\right)/\left(6u^{2}+13.0u+2\right)\;\;\text{for}\;\;r_{ij}\leq r_{\text{int}}$$

In Equation (13) φ stands for the *local* volume fraction of oil around a central particle “*i*”, and

(15) $$u=\left(r_{ij}-R_{i}-R_{j}\right)/{\tilde{R}}_{0}$$

where *R_i_* is the radius of particle *i*, and *R͂*_0_ a radius of a reference. The third term on the right hand side of Equation (12) gives the random deviates of the moving particles. The stochastic vector $\overset{\to }{G}\text{auss}$ stands for a set of random numbers generated by the Box-Muller algorithm. The characteristic mean square displacement of the Brownian movement is obtained multiplying each random deviate by $\sqrt{2D_{\text{eff},i}\left(d,\varphi \right)\Delta t}$.

In ESS, non-deformable drops coalesce if the distance between their centres of mass gets smaller than the sum of their radii:

(16) $$r_{\;ij}\;<\;\;R_{i}+R_{j}$$

When this occurs a new drop is created at the centre of mass of the coalescing drops. The radius of the new drop results from the conservation of volume:

(17) $$R_{new}=\sqrt[{3}]{R_{i}^{3}+R_{j}^{3}}$$

### 3.2. Interaction Forces and the surface excess

In ESS the attractive force between the drops is determined by the effective Hamaker constant of two oil drops separated by water [32–33]. This constant (*A_H_*) is often of the order of 10^−21^ J for hydrocarbons and lattices. In the case of Bitumen some old experimental evidence indicated a value of *A_H_* ∼ 10^−19^ J, but recent evaluations suggest a much lower value (*A_H_* ∼ 10^−21^ J).

The van der Waals potential for two spherical drops of different radius (*R_1_*, *R_2_*) is equal to [32]:

(18) $$V_{A}=V_{vdW}=\left(-A_{H}/12\right)\left\lfloor y/\left(x^{2}+xy+x\right)+y/\left(x^{2}+xy+x+y\right)+2\text{ln}\left(\left(x^{2}+xy+x\right)/\left(x^{2}+xy+x+y\right)\right)\right\rfloor$$

here: *x* = *h*/2*R*_1_, *y* = *R*_2_/*R*_1_, and *h* = *r_ij_* −*R*_1_−*R*_2_.

There is always an attractive force between two drops of similar composition suspended in aqueous media. However, the repulsive force depends on the characteristics of the O/W interface and the type of surfactant adsorbed. Oil drops often exhibit an electrostatic surface potential between −10 mV (octacosane [34]) and −35 mV (benzene [35]) when suspended in water. This charge originates from the preferential adsorption of OH^−^ ions to the oil/water interface. If this is the case, an initial value of the surface charge per unit area (σ_0_) can be introduced as input in order to reproduce the total value of the surface charge *σ_T_* in the absence of surfactants.

When ionic surfactants are present, they add their charge to the initial surface charge of the drop (σ*_0_*):

(19) $$\sigma_{T}=\sigma_{0}+z_{s}\;e\;N_{i}/\left(4\;\pi \;R_{i}^{2}\right)\;=\;\sigma_{0}+A_{i}z_{s}e\;\text{Γ}/\left(4\;\pi \;R_{i}^{2}\right)\;=\;\sigma_{0}+z_{s}e\;\text{Γ}$$

Here *N_i_*, *A_i_*, *z_s_, e* stand for the number of surfactant molecules attached to drop *i,* the area of the drop: *A_i_* = *4 π* $R_{i}^{2}$, the effective valence of a surfactant, and the unit of electrostatic charge (1.6 × 10^−19^ Coul.). Γ stands for number of surfactant molecules per unit area at the oil/water interface. The interfacial area of one surfactant molecule at maximum packing (*A_s_* = $\Gamma_{\text{max}}^{-1}$) is typically of the order of 50 Å^2^ [36]. It depends on the adsorption time and on the way the surfactant partition between the immiscible phases and the interface.

The effective charge of an ionic surfactant molecule (*q* = *z_s_* *e*) can be calculated from the zeta potential (*ζ*) of a drop saturated with surfactant molecules. In the most usual situation *z_s_* is varied until the experimental value of *ζ* is reproduced. This calculation is done assuming that *σ_0_* = 0. In this case, the effective charge of the surfactant contains the contribution coming from the hydration layer. It is also possible to use a finite value of *σ_0_* to reproduce the surface potential of an oil drop in water, and then vary *z_s_* to fit the value of *ζ*. The value of *z_s_* results from apportioning the effective charge of a drop to a discrete number of surfactant molecules (0.17 ≤ *z_s_* ≤0.23 [37]).

The estimation of *q* requires knowledge of the maximum number of surfactant molecules in each drop. This number is equal to:

(20) $$N_{s,i}^{\text{max}}\;=\;4\;\pi \;R_{i}^{2}/A_{s}$$

The current version of the program contains four analytical expressions for the calculation of the electrostatic force [38–42]. The formalism of Sader et al. [39–41] was used in previous simulations of non-deformable drops. According to these authors, the electrostatic potential between two spherical drops is given by Equation (21):

(21) $$V_{E}/k_{B}T\;=\;\left\lfloor C\;B\left(i\right)B\left(j\right)/r_{ij}\right\rfloor \;R_{i}\;R_{j}\;\text{exp}\;\kappa \;h$$

where:

(22) $$C=4\;\pi \;k_{B}\;T\;\varepsilon \;\varepsilon_{0}/e^{2}$$

(23) $$B\left(i\right)=\left({\text{Φ}}_{P}+4\;\bar{\text{γ}}\;\text{Ω}\;\kappa \;R_{i}\right)/\left(1+\;\text{Ω}\;\kappa \;R_{i}\right)$$

(24) $$\bar{\gamma }\;=\text{tanh}\left({\text{Φ}}_{P}/4\right)$$

(25) $$\text{Ω}=\left({\text{Φ}}_{P}-4\bar{\gamma }\right)/\left(2{\bar{\gamma }}^{3}\right)$$

In Equations (21)–(25), *ɛ*_0_ is the permittivity of vacumm, *ɛ* the dielectric constant, *κ*^−1^ the Debye length, and Φ*_P_* = Ψ_0_ *e/k_B_* *T* the reduced electrostatic potential of the particle at its surface. The relation between the surface charge and the surface potential is given by:

(26) $$\sigma_{T}\;e/\kappa \;\varepsilon \;k_{B}\;T\;=\;{\text{Φ}}_{P}+\;{\text{Φ}}_{P}/\kappa \;R_{i}-\kappa \;R_{i}{\left(2\text{sinh}\left({\text{Φ}}_{P}/2\right)-{\text{Φ}}_{P}\right)}^{2}/\bar{Q}$$

where:

(27) $$\bar{Q}\;=\;4\text{tanh}\left({\text{Φ}}_{P}/4\right)-{\text{Φ}}_{P}-\kappa \;R_{i}\left[2\text{sinh}\left(\Phi_{P}/4\right)-{\text{Φ}}_{P}\right]$$

Notice that knowledge of *σ_T_* and the ionic strength of the solution allow the numerical evaluation of the surface potential from Equation (26), as well as the rest of the variables (Equations (22)–(25)) of the potential (Equation (21)). Since the ionic strength is set by the experimental conditions, and *σ_T_* depends on the surface excess, only Γ is required in order to calculate the electrostatic potential between suspended drops.

Surfactants can be ionic, non-ionic, zwitterionic, and have a complex molecular structure. Hence, the interaction potential between the drops can vary amply. The program of ESS includes expressions for van der Waals, electrostatic, oscillatory, depletion, hydration, and steric forces.

In the case of non-ionic surfactants the stabilization force between emulsion drops is assumed to be steric [43–44]. Steric forces are composed of an osmotic (mixing) contribution and an elastic one:

(28) $$V_{steric}=\;V_{osm}\left(h\right)\;+\;V_{elast}\left(h\right)$$

where *h* stands for the minimum distance between the surfaces of the drops. The mixing contribution is generated by the cross linking of the hydrophilic chains of the surfactants of each drop. The elastic force comes from the drastic modification of the surfactant conformation at close distances of separation between the particles. We developed expressions for both types of contributions modifying the equations reported by Vincent [45] and Bagchi [46]. On the one hand, Vincent used simple analytical formulas to describe the distribution of polymer monomers perpendicular to a planar interface. Following he used the Derjaguin approximation [47] to evaluate the expression of the free energy of mixing deduced by Flory and Krigbaum [48], for two spheres stabilized with polymer molecules. On the other hand, Bagchi calculated the exact volume of overlap between the polymer layers of two colliding drops, but used the Flory-Huggins [49] expression for the calculation of the free energy. Lozsán et al. [43] showed that the methodology of Vincent with the exact calculation of the volume of overlap is equivalent to the methodology of Bagchi if the free energy is evaluated with the expression of Flory-Krigbaum.

The ESS code has several expressions for the calculation of the steric interactions [43] including the expression of De Gennes [50]. An example of these expressions is given by Equation (29) for distances between δ < *h* < 2δ [43–44]:

(29) $$\begin{array}{l} V_{osm}\left(h\right)\;=\;\left(4k_{B}T/3V_{w}\right)\left[{\bar{\varphi }}_{i}{\bar{\varphi }}_{j}\right]\left(1/2-\chi \right){\left(\delta -h/2\right)}^{2}\;x\\ \;\;\;\;\;\;\left(3\left(R_{i}+R_{j}\right)/2\;+\;2\delta \;+\;h/2\;-3{\left(R_{i}-R_{j}\right)}^{2}/2\left(h+R_{i}+R_{j}\right)\right) \end{array}$$

In Equation (29) *V_w_* stands for the molar volume of the continuous phase, χ is the Flory-Huggins solvency parameter, and *φ̄_i_* stands for the volume fraction of surfactant molecules in the interfacial layer of drop *i*. In order to estimate *φ̄_i_* it is usually assumed that the hydrophobic chain of the surfactant is dissolved in the oil phase, and only its hydrophilic chain lies in the outer region of the drop. If the density of hydrophilic chains in the interfacial layer is assumed to be constant, *φ̄_i_* can be expressed in terms of the surface excess [44]:

(30) $${\bar{\varphi }}_{i}\;=\;3\;R_{i}\;\text{Γ}/\rho_{2}\left({\left(R_{i}+\delta \right)}^{3}-R_{i}^{3}\right)$$

where: *ρ_2_* stand for the density of the hydrophilic chain.

As in the case of the electrostatic potential, the steric potential depends on some parameters and is a function of the surface excess.

### 3.3. Surfactant Distribution (Evaluation of the surface excess)

The value of the surface excess of a surfactant at the interface of emulsion drops cannot be measured directly. It is extrapolated from the variation of the interfacial tension in systems with a macroscopic O/W interface. Unfortunately, emulsions are constantly evolving, continuously changing their drop size distribution and total interfacial area, *A_T_*:

(31) $$A_{T}\;=\;\sum_{i}A_{i}$$

As a result, the surface excess is not constant and the interaction potential between the drops changes as a function of time.

Ionic surfactants are not soluble in the oil phase. They can migrate to the oil phase in the form of inverse micelles if the salt concentration of the system is unusually high. In the typical case, ionic surfactants adsorb to the O/W interface before they form micelles. The amount of surfactant required for the complete coverage of the interface can be larger or equal to the critical micelle concentration (CMC). It varies with the volume fraction of oil (φ) and *A_T_*. The larger the values of φ and *A_T_*, the higher the surfactant concentration required for the complete coverage of the drops.

In the case of non-ionic surfactants the situation is more unpredictable. The partition of non-ionic molecules depends on the affinity of its lipophilic and hydrophilic moieties for the oil and water phases. For example, the partition coefficient of alkylphenol oligomers between water and n-alkanes is equal to [51]:

(32) $$\text{log}\;K_{m}\;=\;-3.75\;+0.45\;m\;-0.0425\;SACN\;+\;0.03\;ACN$$

where *m* is the number of ethylene oxide units in the surfactant molecule, *SACN* is the number of carbon atoms in the alkyl chain of the surfactant, and *ACN* is the number of carbon atoms in the n-alkane molecule. The adsorption of surfactant molecules depends on time and on several formulation variables like the number of methylene groups of the oil molecule (Equation (32)), the salt concentration, the presence of alcohol molecules in the system, etc. Furthermore, adsorption can be reversible or irreversible [52–53].

The routines of surfactant distribution attempt to recreate the most common experimental situations. The strategy of ESS is to apportion the surfactants to the interfaces of the drops in such a way that it reproduces the variation of the surface excess in the experimental system. Consequently, only the movement of the drops is considered explicitly in Equation (12).

Some of the routines for surfactant distribution have a formal theoretical background [54–55]. Some others are only practical approximations to the very complex problem of surfactant diffusion and adsorption [56–57]. These routines resemble the cases of Homogeneous and Non-homogeneous surfactant distributions. The effect of non-homogeneous distributions results from an incomplete mixing of the emulsion components. Most recent studies concern the cases in which the surfactant is evenly distributed among the surfaces of the drops. Three examples of these strategies are given below.

#### 3.3.1. Homogeneous Surfactant Distribution with fast and irreversible surfactant adsorption

This is the simplest methodology. It consists in ascribing to each drop a number of surfactant molecules (*N_s_*_,_*_i_*) proportional to its interfacial area.

(33) $$N_{s,i}\;=\;N_{s,T}\left(A_{i}/\sum_{i}A_{i}\right)\;\;=\;\;N_{s,T}\;A_{i}/A_{T}\;\left(N_{s,i}\leq \;\;N_{s,i}^{\text{max}}\right)$$

Here *N_s,T_* stands for the total number of surfactant molecules in the system. Equation (33) can always be applied regardless of the total surfactant concentration available. It resembles the cases in which: (a) the surfactant adsorption is very fast in comparison to the collision of drops; (b) the mixing conditions are homogeneous; (c) the adsorption is irreversible.

If the number of drops decreases as a consequence of coalescence, Equation (33) can be used to recalculate the number of surfactant molecules adsorbed to the remaining drops. The number of surfactant molecules of each drop increases as the calculation progresses because the total interfacial area of the emulsion decreases as the drops coalesce. However, the interfacial area of one surfactant molecule cannot be lower than its minimum cross-sectional area at the O/W interface (*A_s_*). Hence, *N_s,i_* cannot exceed $N_{s,i}^{\text{max}}$ = *A_i_/A_s_*. If the number of surfactants in the system surpass the amount required for the complete coverage of all the drops, the value of *N_s,i_* is set equal to $N_{s,i}^{\text{max}}$.

#### 3.3.2. Homogeneous Surfactant Distribution with time-dependent surfactant adsorption

In the case that the adsorption is basically controlled by the diffusion of surfactant molecules from the bulk to the subsurface [58], the surface excess is a function of the total surfactant concentration *C_T_,* the diffusion constant of the surfactant *D_s_*, and the time:

(34) $$\text{Γ}\left(t\right)=\;\;2C_{T}\;\;{\left(D_{s}\;\;t/\pi \right)}^{1/2}$$

The mechanism of surfactant adsorption can be very involved, including barriers of adsorption, reorientation at the surface, etc. However, Liggieri et al. [59] demonstrated that most process of mixed adsorption kinetics can be reformulated in terms of a diffusion controlled mechanism (Equation (34)) if *D_s_* is substituted by an “apparent” diffusion constant (*D_app_*). Furthermore, this equation is also compatible with the findings of Hua and Rosen [60–61] for a large number of surfactants with different molecular structures. According to these authors, the surface tension of most surfactants shows four characteristic regions of change known as: the induction period, the fast-fall region, the mesoequilibrium region and the equilibrium region. The time required for the surface pressure to drop to half its value at mesoequilibrium, is found to follow Equation (35). This equation allows estimating the value of *D_app_* required for the evaluation of Equation (34).

(35) $$\text{log}(t)\;=\;2\text{log}\left(\text{Γ}\left(t\right)/C_{T}\right)\;+\;\text{log}{\left(\pi /4D_{app}\right)}^{1/2}$$

The routine of time dependent surfactant adsorption uses *D_app_*, and *C_T_* as input, estimating the value of the surface excess from Γ(*t*) = *2 C_T_* (*D_app_* *t/π*)^1/2^. The number of surfactant molecules of drop *i* is calculated according to Equation (36) [55]:

(36) $$N_{s,i}\;=\;\;A_{i}/A_{s}\left(t\right)\;=\;\;A_{i}\;\text{Γ}\left(t\right)\;\;=\;\;4\pi \;R_{i}^{2}\;\text{Γ}\left(t\right)$$

Here *A_s_*(*t*) stands for an “effective” area per surfactant molecule at the oil/water interface. As Γ(*t*) increases, *A_s_*(*t*) decreases, increasing the number of surfactant molecules adsorbed to each drop.

#### 3.3.3. Equilibrium Surfactant Distribution (Gibbs)

Equilibrium isotherms are only attained after long periods of time. In this routine we assume that: a) the adsorption of surfactant is very fast, and b) equilibrium adsorption is obtained instantaneously.

Gibbs isotherm only applies to that range of surfactant concentration between the beginning of the decrease of the interfacial tension *C_T_* = *C_1_*, and the Critical Micelle Concentration (CMC). When *C_T_* ≥ C_cmc_ the maximum number of surfactants per drop is already adsorbed: *N_s,i_* = $N_{s,i}^{\text{max}}$ (Equation (20)). When *C_T_*≤*C_1_* the change in the interfacial tension often shows a small maximum. We disregard this feature of the experimental curve. Instead we approximate the low concentration limit either by a) using an additional Gibbs isotherm between *C_T_* = 0 and *C_T_* = *C_1_*; or b) calculating an average surface excess including the point *C_T_* = 0, γ = γ_0_.

In terms of finite differentials the equation of Gibbs is equal to:

(37) $$\gamma_{2}\;\;=\;\;\gamma_{1}\;\;-ck_{B}\;T\;\tilde{\text{Γ}}\;\text{log}\left(C_{2}/C_{1}\right)$$

where c = 1 or 2 depending on the type of surfactant and the ionic strength of the solution. Assuming proportionality between the suggested value of the tension and the number of surfactant molecules adsorbed:

(38) $$\begin{array}{cc} \begin{array}{cc} \gamma_{2}\;\;=\;\;\gamma_{1} & +\;\left(\gamma_{2}-\gamma_{1}\right) \end{array}\;\left[N_{s,i}/N_{s,i}^{\text{max}}\right] & \left(\gamma_{2}\;<\;\gamma_{1}\right) \end{array}$$

Equation (38) is very convenient because it expresses the tension in terms of the surfactant population of each drop. According to Equations (37)–(38):

(39) $$\begin{array}{cc} N_{s,i}\;=\;\left(-c\;k_{B}\;T\;\tilde{\text{Γ}}\;N_{s,i}^{\text{max}}/\left[\gamma_{cmc}-\gamma_{1}\right]\right)\text{log}\left(C_{T}/C_{1}\right) & \gamma_{1}\;>\;\gamma_{cmc} \end{array}$$

Equation (39) gives the number of surfactants attached to drop *i* as a function of the total surfactant concentration in the system. Here, *γ*_1_ stands for the value of the interfacial tension at *C_1_*. The value of Γ*_i_* is obtained from Equation (39) dividing *N_s,i_* by *A_i_*. Notice that there might be cases in which the number of surfactant molecules in the system might not be enough to cover the drops according to Equation (39). In those cases a homogeneous surfactant distribution is applied (Equation (33)) despite the initial selection of the Gibbs routine.

### 3.4. Ostwald Ripening

Although a large number of numerical techniques are available for the simulation of Ostwald ripening [62–66] they are based on population balance equations and cannot be incorporated into the algorithm of ESS. In order to simulate the process of Ostwald ripening we included the algorithm of De Smet *et al*. [67–69] in our ESS code. The fundamental equation of this method is derived from the Fick’s law and the Kelvin’s equation assuming α≪ *R_i_* :

(40) $$dn_{o,i}\left(t\right)/dt\;=\;4\;\pi \;{\tilde{D}}_{oil}\;C\left(\infty \right)\alpha \;\left(R_{i}\;\left(t\right)/R_{c}\;\left(t\right)-\;1\right)$$

Here, *n_o,i_* stands for the number of molecules of oil in particle *i. D͂_oil_* refers to the diffusion constant of an oil molecule. *C*(∞) stands for the aqueous solubility of the oil in the presence of a planar O/W interface. *R_c_* is the critical radius of the emulsion equal to [70]:

(41) $$R_{c}\;\left(t\right)\;\;=\;\;\frac{1}{N}\sum_{i}R_{i}$$

Here *N* is the total number of drops. Parameter α is the so called capillary length, defined as:

(42) $$\alpha \;=\;2\gamma \;V_{m}/\hat{R}T$$

In Equation (42) *V_m_* is the molar volume of the oil and *R̂* the gas constant. Defining:

(43) $$P_{i}\left(t\right)=R_{i}\;\left(t\right)/R_{c}\;\left(t\right)-\;1$$

and:

(44) $$M\;=\;4\pi \;D_{m}\;C\left(\infty \right)\alpha \;\text{Δ}t,$$

A simple equation for the exchange of oil molecules is obtained:

(45) $$n_{o,i}\left(t+\;\text{Δ}t\right)\;=\;\;n_{o,i}\left(t\right)\;\;+\;\;M\left(t\right)\;P_{i}\left(t\right)$$

At any step of the simulation there exists a critical radius *R_c_* of the emulsion. Particles with *R_i_* < *R_c_*, dissolve while particles with *R_i_* > *R_c_* grow. Particles with the same radius as the critical radius *R_i_*_=_ *R_c_* preserve their size. The number of molecules exchanged by particle *i* is equal to the product *M* (*t*) *P_i_* (*t*).

In ESS the value of *M*(*t*) is set once the time step of the simulation is chosen, *M* (*t*) = *M*. When the smallest particle *i* = *small* contains fewer molecules than the number it should lose according to Equation (45), *M(t)* is substituted by:

(46) $$M\left(t\right)=n_{o,small}/M$$

Recently we implemented a new procedure to avoid a substantial decrease in the number of particles [71]. The simulation starts from a given Drop Size Distribution (DSD) of *N_0_* drops, and evolves until it reaches *N*(*t* = *t*′) = *N_1_* = 200. At this point, a new Drop Size Distribution (DSD) with *N* = *N_0_* drops is built. This can be achieved approximating the probability distribution of particle sizes by the relative number of drops of each size existing at time *t* = *t*′:

(47) $$\tilde{P}\left(R_{i}\right)\;=\;N\left(R_{i},t^{\prime }\right)/N_{1}$$

where *N*(*R_i_*,*t*′) is the number of drops with radius *R_i_* existing at time *t*′. Thus, the number of drops of size *R_i_* in the new distribution, *N*′ (*R_i_*, *t*′), is equal to:

(48) $$N^{\prime }\left(R_{i},t^{\prime }\right)\;\;=\;\;N_{0}\;N\left(R_{i},t^{\prime }\right)/N_{1}\;\;=\;\;N\left(R_{i},t^{\prime }\right)\left(N_{0}/N_{1}\right)$$

Use of Equations (47)–(48) produces a new DSD with *N_0_* drops that exactly matches the old one. The auxiliary code also calculates the new size of the simulation box, which is required in order to preserve the initial volume fraction of oil with the new set of particles. Once the input file is modified and the new distribution read from an external file, the program generates a new set of co-ordinates for the new particles. As in the beginning of the simulation the particles are distributed at random avoiding overlap. At this point the code resumes the calculation of the main cycle (see below).

### 3.5. The Algorithm of ESS

**Figure 2.**
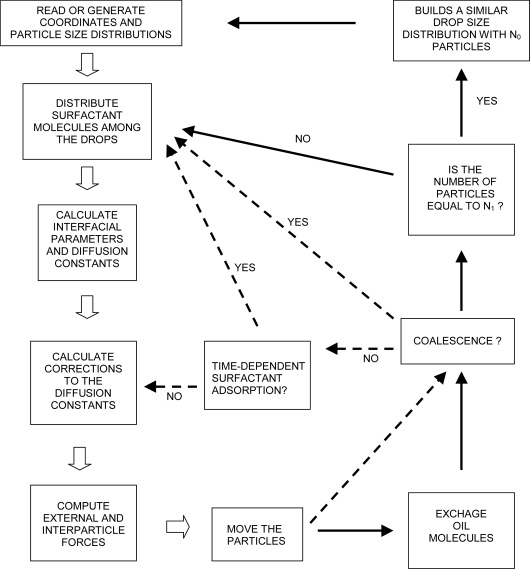
Flowchart of Emulsion Stability Simulations.

The algorithm for ESS is shown in Figure 2 in the form of a flowchart.

At the beginning of the simulation the code reads or generates a drop size distribution and a set of co-ordinates for each particle. Additionally, the time step(s) of the simulation (single or double) must be specified. A combination of a small time step and a large one is used for the calculation of dilute dispersions [72]. For this purpose a distance of closest approach (d_min_) must also be selected. The maximum range of the interaction potential usually approximates this distance (50 nm ≤ d_min_ ≤ 100 nm). A double time-step calculation implies the use of a longer time step (Δ*t_L_*) when the particles are far apart from each other, *r_ij_* > d_min_. If the distance between two particles becomes equal or lower than d_min_, all particles are returned to their previous positions, and a small time step (Δ*t_S_*) is used in Equation (12) until ∑Δ*t_S_*=Δ*t_L_*. This technique is efficient in those cases where the repulsive potential does not prevent coalescence. Otherwise the drops aggregate making *r_ij_* always lower than d_min_. This causes the continuous use of Δ*t_S_* after the first floc is formed.

After the creation of the DSD, the program distributes the surfactant molecules among the drops. There are 11 routines to cover the most common experimental situations.

At this point the interfacial tension of each drop can be determined. The calculation of the diffusion constants is also executed here, because some of its expressions depend on interfacial parameters. First, the program assigns the diffusion constant of Stokes to all particles according to their radius. Second it makes the corrections necessary to account for the effect of the interfacial properties and hydrodynamic interactions on the diffusion constant ( $f_{\text{corr}}^{\left(1\right)}$ and $f_{\text{corr}}^{\left(2\right)}$).

Following, the forces are calculated and the drops are moved according to Equation (12). If the Ostwald ripening mode is selected, the oil molecules are exchanged at this point, and the program follows the path indicated by the thin solid arrows in Figure 2. Otherwise the program follows the path indicated by the thin broken-line arrows. In the latter case, the program checks for coalescence after the particles are moved. In the former case the program checks for coalescence after the exchange of oil molecules takes place.

If coalescence occurs, the total interfacial area of the emulsion changes along with the volume of one (or several) drop(s). Therefore the surfactant population had to be redistributed among the drops. The same occurs if Ostwald ripening happens. However, in the latter case a minimum number of drops must be maintained. Therefore, the program checks if the remaining number of drops is higher than a fixed value (*N*(*t* = *t*′) > *N_1_*). If this is the case, the program proceeds with the redistribution of surfactant molecules. Otherwise, the program stops. An auxiliary program is used to build a new drop size distribution with *N* = *N_0_* = *N* (*t* = 0) drops. This program also calculates the new size of the box necessary to keep the volume fraction of oil constant with the new set of particles. The new distribution is read from a file by the program of ESS. The co-ordinates of the new particles are then generated and the main cycle of ESS continues.

If the Ostwald ripening process is not selected, the program neither exchange oil molecules nor does it re-builds the drop size distribution when *N*(*t* = *t*′) ≤ *N_1_*. In this case the code follows different routes depending on the outcome of the coalescence check. If coalescence occurs it is necessary to reassign the surfactant population. If it does not occur there are two possibilities. If one of the routines of time-dependent adsorption is used, it must recalculate the surfactant population anyway. If this is not the case, the program proceeds with the evaluation of the hydrodynamic interactions and a new cycle begins.

It is important to remark at this point that the change of the number of particles during the simulations is only equal to the variation of the number of aggregates *in the absence of a repulsive force.* This was demonstrated quantitatively in Ref [73].

Whenever a repulsive barrier is present, the change in the number of aggregates as a function of time has to be calculated at the end of the simulation using a different program [21, 73–74]. This additional code uses as input the positions of the particles produced during the simulation and a fixed flocculation distance. The flocculation distance is usually approximated by the position of the secondary minimum of the interaction potential. When these computations are finished, statistical data regarding aggregates, flocs, radius of gyration, etc, is obtained.

## 4. Results

The general results of the simulations can be classified into three categories depending on the total surfactant concentration of the system [74].

### 4.1. Low surfactant concentration

If the surfactant concentration is not enough to stabilize the initial drop size distribution, drops coalesce as soon as they collide with each other. Hence, the change in the number of particles is equal to the change in the number of aggregates [73]. In this case, the systems follow the dynamics of Smoluchowski, in the sense that the number of aggregates changes as predicted by Equation (10) with *W* = 1 [72–73]:

(49) $$n\left(t\right)\;\;=\;\;n_{0}/\left(1+k_{f}^{fast}n_{0}\;t\right)$$

The value of $k_{f}^{\text{fast}}$ depends on the mean free path between the drops and their attractive force. For volume fractions between 10^−5^ ≤ *φ* ≤ 0.30 and a Hamaker constant *A_H_* ∼ 10^−21^ J [72]:

(50) $$k_{f}^{fast}\;\;=\;\;\left(6.44\;x\;10^{-18}\right)\text{exp}\left(8.04\;\varphi \right)\;\;{\text{m}}^{3}/\text{s}$$

In the case of *A_H_* ∼ 10^−19^ J:

(51) $$k_{f}^{fast}\;\;=\;\;\left(9.55\;x\;10^{-18}\right)\text{exp}\left(15.41\;\varphi \right)\;\;{\text{m}}^{3}/\text{s}$$

Notice that the value of $k_{f}^{\text{fast}}$ differs from the theoretical estimation of Smoluchowski (*k_S_* = 4*k_B_* *T/*3*η ∼* 6.11 × 10^−18^ m^3^/s) based on the Brownian motion of the particles only.

The attractive force between the drops increases the value of $k_{f}^{\text{fast}}$ while the hydrodynamic forces decrease it. For *A_H_* ∼ 10^−21^ J the effect of the attractive potential is small and the hydrodynamic interactions dominate (see Figure 8 in Ref. [29]). These are the cases of hydrocarbon-in-water emulsions and latex dispersions. For *A_H_* ∼ 10^−19^ J (polar oils, metal salts) the attractive force dominates. In either case, the effect of the volume fraction remains. As *φ* increases the mean free path between the drops decreases. This diminishes the time of diffusion between the collisions of the particles, increasing the value of $k_{f}^{\text{fast}}$.

Notice that Equation (49) was intended to explain the phenomenon of irreversible flocculation of *solid* particles. Danov *et al.* [75] demonstrated that Equation (49) also applies to emulsions subject to the simultaneous processes of flocculation and coalescence. For this demonstration no distinction was made between aggregates formed by the collision of smaller flocs, and those of the same size resulting from the partial coalescence of flocculated drops. Similarly, no distinction was made between single particles formed through coalescence, and those initially present at *t* = 0. These are the same assumptions we use for determining the variation of the number of the aggregates as a function of time. Danov et al. also supposed a constant collision kernel, but did not make any specific hypothesis regarding the coalescence rates.

The fact that Equation (49) holds for coalescing emulsions is related to the suppositions followed by Smoluchowski for determining the flocculation rate. He pictured the case in which one particle was fixed in space while the others collided with it as a consequence of a gradient of concentration. This gradient was established as soon as t > 0 between the collision radius of the fixed particle and the bulk of the liquid. However, the density of particles at the collision radius was assumed to be equal to zero during the whole aggregation process. Hence, the fixed particle acted as a perfect sink. Every particle that collided with the fixed particle was assumed to disappear at the moment of the collision. Moreover, the collision efficiency of a cluster composed of single particles was estimated using the same procedure, but employing the *average hydrodynamic radius* of the aggregate to approximate its collision radius. As a result of these assumptions, the process of flocculation described by the theory of Smoluchowski “includes” the possible occurrence of *instantaneous* coalescence. Instantaneous meaning that the time required for coalescence is negligible in comparison to the time required for flocculation.

Inhomogeneous surfactant distributions as well as reversible adsorption, also lead to fast aggregation and to the coalescence of drops, favouring the validity of Equation (49) [54]. The same occurs if the surfactant does not adsorb rapidly to the O/W interface (*D_app_* < 10^−12^ m^2^/s) [55].

**Figure 3.**
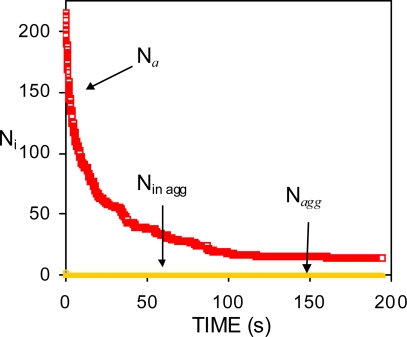
Smoluchowskian decrease of the number of aggregates as a function of time. Subscripts “*a*”, “in agg” and “*agg*” stand for: the number of aggregates plus single particles (*N_a_*), the total number of particles in aggregates (*N*_in agg_), and the number of flocs (*N*_agg_), respectively (*A_H_* = 1.24 × 10^−19^ J).

The data of Figure 3 corresponds to system #12 in Refs. [19, 73]. The surfactant concentration in the system (*C_s_* = 10^−5^ M) is not enough to prevent the coalescence of drops during the course of the simulation. Hence, the number of single particles is equal to the *total* number of aggregates (*N_a_*). The number of aggregates constituted by two or more particles (*N_agg_*) is zero, as well as the number of particles *in* aggregates (*N*_in agg_).

In general, the initial interfacial area of an emulsion is higher than the one that can be stabilised with the surfactant concentration available. In this case a Smoluchowskian drop in the number of particles occurs at short times. In the preparation of emulsions, chemical methods usually employ a larger surfactant concentration than mechanical ones. This favours the formation of a repulsive barrier as soon as the drops are formed. In this case, the dynamic of aggregation is not expected to follow Equation (49).

### 4.2. Intermediate surfactant concentration. Insufficient surfactant molecules to prevent the initial coalescence of drops

**Figure 4.**
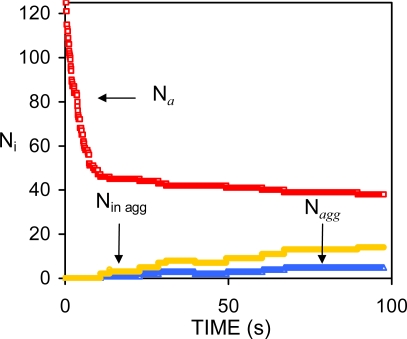
Behavior of system #13 from Refs. [19, 73–74]. The subscripts are the same of Figure 3. (φ = 0.22, *C_s_* = 8.65 × 10^−5^ M, *A_H_* = 1.24 × 10^−19^ J).

According to ESS of non-deformable droplets, the variation of the number of aggregates in the presence of an appreciable repulsive barrier conforms to a remarkably simple expression [73]:

(52) $$n_{a}\;\;=\;\;n_{0}\left[A/\left(1+k_{1}\;n_{0}\;t\right)\;\;+\;\;B\;\text{exp}\left(-k_{2}\;n_{0}\;t\right)\right]$$

where *A, B, k_1_* and *k_2_* are constants and *B* = 1 -* A.* The second term in Equation (52) results from the consideration of the coalescence rate as a first order process, depending on the number of flocculated doublets. We used a Smoluchowskian term to represent flocculation and an exponential term to represent coalescence. Hence, *k_1_* and *k_2_* were formerly ascribed to the flocculation rate ( $k_{f}^{\text{slow}}$) and the coalescence rate (*k_c_*)*,* respectively [73]. Coefficients *A* and *B* measure the extent of these two processes during the simulation. The curve described by Equation (52) generally shows a pronounced initial decrease followed by a much slower change in the number of aggregates per unit volume (*n_a_* = *N_a_*/*V*). See Figure 4. The initial decrease corresponds to the combined processes of flocculation and coalescence (FC period). During the FC interval, the total interfacial area of the emulsion decreases. This causes a substantial redistribution of surfactant molecules amongst the remaining drops if a homogeneous surfactant distribution is assumed. As a result there is a progressive increase in the repulsive potential between the drops, which slows down both destabilisation processes.

Equation (52) was able to reproduce the behaviour of 34 simulations, including different DSDs, surfactant concentrations, number of particles, volume fractions (0.01 ≤ *φ* ≤ 0.30), and spatial distributions. This is remarkable since the structure of the aggregates and their spatial distributions changed considerably from one system to another.

**Figure 5.**
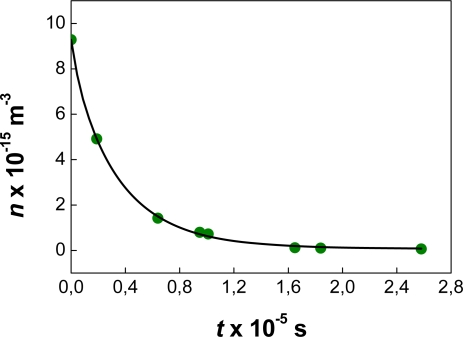
Variation of the number of particles as a function of time for a dodecane in water nano-emulsion stabilized with Brij 30 (*φ* = 0.20).

We have also compared the prediction of Equation (52) with some unpublished experimental data on dodecane-in-water nanoemulsions (*φ* = 0.20, *T* = 25 C) obtaining good results. For this purpose the average radius of the emulsion was measured as a function of time. The number of *particles* was estimated dividing the total volume of oil by the average volume of a drop at each time. Based on the results of the previous section (*4.1*)*,* we approximated the number of aggregates by the number of particles *during the FC period,* and used Equation (52) to fit the results. The fitting is shown in Figure 5 along with the experimental data (green circles). Notice that the experimental curve shows a sharp decrease in the FC rates after a pronounced initial drop in the number of *particles.* Equation (52) also appears to fit the data beyond the FC interval.

Series expansions of the exponential term and the flocculation function of Equation (52) around *t* = 0, showed that these two functions are very similar. In fact, it is possible to exchange the pair of coefficients corresponding to each process (*A*, *k_1_*) ↔ (*B*, *k_2_*) and obtain a fitting of similar quality. This demonstrated that the processes of flocculation and coalescence are mixed in the kinetic rates of Equation (52). Moreover, it was also observed that Equation (52) was able to fit a terminal aggregation stage in those systems in which the number of particles stabilises after a certain period of time.

**Figure 6.**
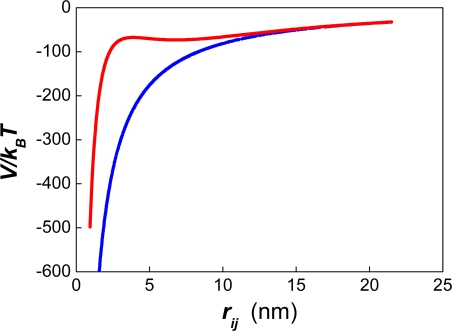
DLVO potential (red line) between two spherical drops of bitumen (*R_i_* = 0.39 μm) with a surface charge of σ = 13.95 mCoul/m^2^ (Ψ_0_ ∼44 mV, *A_H_* = 1.24 × 10^−19^ J). The ionic strength of the solution is equal to 1.4 × 10^−2^ M. The van der Waals potential is shown in blue.

**Figure 7.**
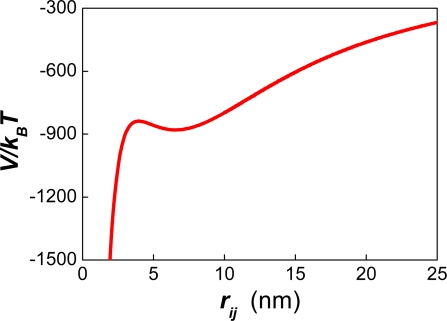
Potential energy between two spherical drops of bitumen (*R_i_* = 3.9 μm) with a surface charge of σ = 13.95 mCoul/m^2^ (Ψ_0_ ∼44 mV, *A_H_* = 1.24 × 10^−19^ J). The ionic strength of the solution is equal to 1.4 × 10^−2^ M.

It was also found that the interfacial area of the emulsion after ∼200 seconds was proportional to the number of surfactant molecules. The slope of this curve provides a measurement of the area per surfactant molecule that is required (∼ 236 Å^2^) in order to avoid an initial pronounced drop in the number of particles. Using the effective charge of a surfactant molecule (0.21e), the corresponding value of the surface charge can be obtained (σ = 13.95 mCoul/m^2^). The electrostatic surface potential can also be calculated (Ψ_0_ ∼44 mV) using the relation between σ and Ψ (Equation (26)). Unexpectedly, these values do not correspond to a positive magnitude of the potential energy for the referred systems. Instead, they correspond to the appearance of a shoulder at negative values of the potential energy (see Figure 6–7). This shoulder originates the secondary minimum (for which *F_i_* =−∂*V/*∂*r* = 0)*,* along with a small potential barrier. Yet, this barrier occurs at negative values of the interaction potential where an attractive force between the particles exists.

**Figure 8.**
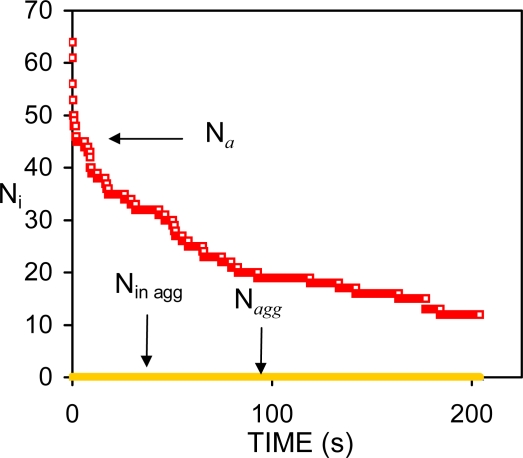
Behavior of system #12 from Refs. [19,73–74]. The subscripts are the same of Figure 3. (φ = 0.16, *C*_s_ = 4.10 × 10^−5^ M, *, A_H_* = 1.24 × 10^−19^ J).

From the variation of *n_a_* vs. *t* (or *N_a_* vs. *t*) in the systems studied (see Figure 4) it is clear that a considerable stabilisation is reached shortly after the FC interval. At this point, several factors may favour the decrease of the flocculation rate. The number of remaining drops after the FC period is smaller and the mean free path between the drops is larger than it was at the beginning of the simulation. Moreover, the diffusion constant of the remaining drops is smaller since it is inversely proportional to the radius of the drops. However, these factors are likely to produce a progressive decrease of the FC rate as is observed in Figure 8. Instead a sharp change in the behaviour of *n_a_* was observed in most systems studied in Ref. [73].

We believe that the drastic change in the slope of *n_a_* vs. *t* is basically caused by the increase of the repulsive force between the remaining particles of the emulsion. In this regard it should be noticed that when the particles execute Brownian motion, their kinetic energy is lost in a short period of time. As a result, the total energy of the drops should approach closely the potential energy curve. Hence, the absence of a driving force at the secondary minimum (*F_i_* =−∂*V/*∂*r* = 0) should slow down considerably the movement of small drops. In this situation a small repulsive force could be enough to prevent primary minimum flocculation (coalescence).

A more conventional effect of the repulsive barrier was observed in the simulation of hexadecane-in-water nanoparticles (*R_i_* = 200 nm) stabilized with nonyl phenol ethoxylated (NPE) surfactants. In these calculations the repulsive potential was assumed to be steric, and the interfacial area of the surfactant molecules was kept constant during the whole simulation. These calculations showed that there is an analytical relationship between the height of the repulsive barrier of the interaction potential and the stability ratio:

(53) $$\text{log}\left(W\right)\;\;=\;\;0.493\left(\text{Δ}V/k_{B}T\;\;-\;\;1\right)$$

This equation is very similar to Equation (9) except for the method of evaluation of Δ*V* and *W*. Equation (9) was deduced from the systematic evaluation of Equation (8) for the case of solid particles suspended in water. Instead Equation (53) was deduced from emulsion stability simulations of liquid drops exposed to flocculation *and coalescence*. In the present case Δ*V* was measured from the base of the secondary minimum up to the top of the potential barrier. In the former case the height of the barrier was measured from *V* = 0. Moreover, Equation (8) was deduced assuming the validity of the formalism of Smoluchowski (Equation (10)). However, the plot *n_a_* vs. *t* does not follow the Equation (10) in the presence of a sizeable repulsive barrier. Instead, we redefined *W* in terms of the half-lifetime of the dispersion:

(54) $$W\;\;=\;\;t_{1/2}^{slow}/t_{1/2}^{fast}$$

Equation (54) results from the inverse proportionality between the half-lifetime and the flocculation rate suggested by Equation (11). However, it does not depend on the particular form of the curve of *n_a_* vs. *t.*

### 4.3. High surfactant concentrations

Equation (8) does not impose any restriction to the value of *W:* the higher the potential barrier the higher the stability ratio. However, according to ESS of non-deformable drops [44], the number of particles of the emulsion is preserved when the repulsive barrier between the drops is higher than 12.7 *k_B_* *T.* In this case the dynamics of the system depend on the depth of the secondary minimum of the interaction potential [21].

According to Verwey and Overbeek [20] a 25*-k_B_T* barrier is necessary for a 7-day stabilization of a concentrated emulsion (*n_0_* *∼* 10^14^ m^−3^). This corresponds to *W* = 10^9^. Much higher values of the stability ratio (10^10^ – 10^17^) are found in the literature published between 1917 and 1940 (see Refs. [21, 76] and citations therein). These high stability ratios mostly correspond to sols of very small particles (between 20 nm and 200 nm). Much lower values of *W* (1 ≤ *W* ≤100) are found for latex suspensions (see Table 1 in Ref [21]).

The wide range of stability ratios reported during the past century is partially caused by the inadequate definition of *W.* The first expression of *W* deduced by Fuchs [24] compared the actual value of the flocculation rate with the theoretical value predicted by Smoluchowski. These stability ratios tend to be high since Smoluchowski did not include the effect of the attractive force in the calculation of $k_{f}^{\text{fast}}$ (*k_S_* =4*k_B_T/*3*η*). However, even when the correct expression of *W* is used (Equation (8)), its value rarely surpasses 1000 for particles between 0.5 μm and a few microns. These low values of *W* do not agree with the fact that most of these particles exhibit a high repulsive barrier against primary minimum flocculation (Equations (9)).

We studied this problem in Ref [21] using several particle sizes and interaction potentials. Varying the surface charge of the particles and the ionic strength of the solution, it was evident that particles between 10 nm and 100 nm usually show very shallow secondary minima or no secondary minima at all. Micron size particles instead show deep secondary minima.

The simulations indicated that the systems with barrier heights higher than 20 *k_B_T* preserve the number of particles (coalescence does not occur *during the extent of the simulation*). In these cases the depth of the secondary minimum determines the evolution of the system. On the one hand, shallow minima do not lead to aggregation [21,74]. Hence, the total number of aggregates (*n_a_*) oscillates frequently around the number of particles indicating the sporadic formation of doublets and their quick dissolution. On the other hand, deep secondary minimum promotes fast flocculation. In this case *irreversible* secondary-minimum flocculation occurs. The random fluctuation of the number of aggregates previously observed with shallow minima does not occur. The particles do not go in and out the secondary minimum as it happened in the former case. The curves of *n_a_* vs. *t* show a progressive decrease as a function of time, as it is usually exhibit in the case of fast aggregation. It is important to realise that deep secondary minimum often occur at high ionic strength, where the repulsive barrier and the secondary minimum are closest to the particle surface.

Recently Kuznar and Elimelech studied the deposition of micrometer-sized particles on a single layer of packed glass beads using a flow-cell [77]. The colloidal particles used had a mean diameter of 4.1 μm. The diameter of the glass spheres was 1 mm. The shapes of the DLVO potential between the particles and the glass beads (Figs. 6–7 in Ref. [77]) were very similar to those reported by our group in Figure 1 of Ref. [21]. In the experiments of Kuznar and Elimelech the depth of the secondary minimum varied from −0.62 *k_B_T* at 1 mM KCl to −21.7 *k_B_T* at 30 mM. The repulsive barrier of the DLVO potential changed from 5029 *k_B_T* (at 10 mM) to 1089 *k_B_T* (at 30 mM). Notice that huge values of *W* result from introducing these potential barriers into Equations (9) or (53). Hence, these high repulsive barriers should prevent the deposition of particles over the glass beads. However, this behaviour was only observed at 0 mM KCl where the highest repulsive barrier occurred. At 100 mM the repulsive barrier was completely screened by the counter ions, and large amounts of latex particles were deposited. These two findings agree with the theory of DLVO. However, between 10 mM and 30 mM increasing amounts of latex particles were deposited. This behaviour completely contradicts the predictions of DLVO. Moreover, when the ionic strength was decreased after the deposition of the particles, most of them were washed out, but some of them remained attached to the glass beads. In a previous article [78] Tufenkji and Elimelech reported deviations from the classical colloid filtration theory in the presence of repulsive DLVO interactions. According to these authors, the fast deposition of microbial particles on porous columns was caused by the combined effect of favourable and unfavourable colloidal interactions. The authors related the deposition rates to the influence of deep secondary minimum in the aggregation process, proposing a dual-deposition model that includes slow and fast aggregation rates.

The experimental evidence described above strongly supports the predictions of Ref. [21]. Hence, it is possible to obtain fast secondary-minimum flocculation in systems of micron-size particles even in the presence of a high repulsive barrier.

**Figure 9.**
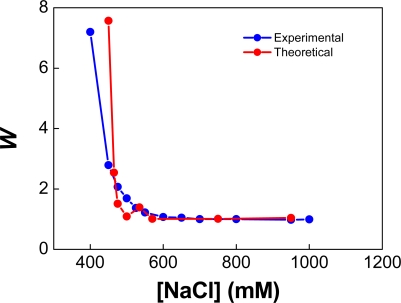
*W* values of 96-nm latex suspensions at several ionic strengths.

Figure 9 shows unpublished experimental data regarding the aggregation of 96-nm sulfonated latex particles. This latex was synthesised by Ms. K. Rahn and Dr. A. Lozsán at the University of Almería, Spain, under the tutorage of Dr. M.S. Romero-Cano. The experimental values of *W* shown in Figure 9 (blue symbols), were evaluated from the initial slope of the absorbance (*Ab*) of latex suspensions as a function of time [79]:

(55) $$W\;\;=\;\;{\left(dAb/dt\right)}_{csalt\;=\;1M}/{\left(dAb/dt\right)}_{csalt}$$

In Equation (55) subscript “*csalt*” stands for the salt concentration of the solution (ionic strength). Theoretical values of *W* were estimated following a methodology similar to the one reported in Ref. [80]. In the present simulations we take advantage of the fact that primary minimum flocculation implies coalescence for non-deformable droplets. Hence, we approximated the *flocculation* time between two latex particles in the experimental set up, by the *coalescence* time between two non-deformable drops with the same interaction potential. The drops were initially located 20 nm apart from each other. The average coalescence time -at each ionic strength- was estimated from 100 simulations, which only differ in the initial value of the random number generator.

(56) $$W\;\;=\;\;t_{c,ave}^{slow}/t_{c,ave}^{fast}$$

For the evaluation of $t_{c,\text{ave}}^{\text{slow}}$ the complete DLVO potential was used. For $t_{c,\text{ave}}^{\text{fast}}$ only the van der Waals contribution was included.

These calculations are the simulation analogue of the numerical evaluation of Equation (8). While many-particle calculations give information about the evolution of the emulsion and the structural characteristics of the aggregates formed, two-particle simulations are restricted to the evaluation of the time required for primary-minimum flocculation. However, these calculation are fast and provide all the information necessary for the evaluation of *W*.

As shown in Figure 9, the value of the stability ratio tends to 1 at high ionic strengths and steeply increases below 570 mM NaCl. The values of *W* produced by the simulations vary non-monotonously around the experimental measurements. This could be caused in part by the low number of calculations used to compute the average values of the coalescence time. Notice also that we could not evaluate the stability ratio corresponding to 400 mM NaCl, because in this case the repulsive barrier was higher than the coalescence threshold of 12.7 *k_B_* *T* (Δ*V* ≥ 12.7 *k_B_* *T*). The calculation corresponding to 400 mM NaCl has been running since several months ago, and not a single coalescence event has been observed.

In our view, the agreement between theory and experiment shown in Figure 9 is reasonable. This suggests that the coalescence times determined by ESS for systems of non-deformable particles should be meaningful.

### 4.4. Time-dependent adsorption

On the one hand a 0.1 g/L solution of sodium dodecyl sulfate (SDS) causes a drop of 15 mN/m in the interfacial tension of a dodecane/water system in a period of 100 s [81]. On the other hand a diluter solution of SDS (C_T_ = 0.014 g/L) achieves the same fall in only 6 s in the presence of 0.1 g/L NaCl. Which chemical condition is more effective for stabilising a dodecane-in-water emulsion considering that the electrostatic interaction is screened in the latter case?

In order to throw some light into this problem, we computed the evolution of a set of oil-in-water emulsions with volume fractions between 0.10 ≤ *φ* ≤ 0.40, apparent diffusion constants in the range 10^−12^ m^2^/s ≤ *D_app_* ≤ 10^−9^ m^2^/s, and two surfactant concentrations *C_T_* = 1 × 10^−4^ M, and *C_T_* = 5 × 10^−4^ M [55]. These parameters cover an ample range of experimental situations. Non ionic surfactants usually show diffusion limited adsorption [58,82]. A typical value for their diffusion constant is 10^−10^ m^2^/s, unless their molecular weight is very large. Ionic surfactants show kinetically limited adsorption [59]. This means that the molecules approaching the interface experience the electrostatic repulsion of those previously adsorbed. Hence, the apparent diffusion constant of these molecules could be substantially lower than 10^−10^ m^2^/s. Furthermore, according to Rosen et al. [60,61] a concentration of 5 × 10^−4^ M is the lowest surfactant concentration required to achieve a 1-second surface tension reduction that does not change much if the surfactant concentration is increased. In these simulations the surfactant was assumed to be ionic. Therefore, the interaction potential between the drops was supposed to be DLVO. Up to our knowledge this was the first off-lattice simulation that took into account the effect of time-dependent surfactant adsorption along with the explicit movement of the drops. The question we addressed in that article was: Under what conditions of *φ*, *C_T_*, and *D_app_* is dynamic adsorption relevant?

The stability of an emulsion depends on the balance between: a) the mean time between the collisions of the drops, and b) the time necessary for a *sufficient* surfactant adsorption.

A close look at Equation (49) indicates that the half lifetime of the emulsion, defined as:

(58) $$t_{1/2}\;\;=\;\;1/k_{f}^{fast}n_{0}\;\;=\;\;t_{f},$$

is also equal to the mean time between collisions (*t* *_f_*). This time depends on the volume fraction of oil, the attractive force between the drops, their initial spatial distribution and the polydispersity of the DSD. The value of *t* *_f_* can be calculated from ESS in the absence of a repulsive force. For monodisperse drop size distributions, *A_H_* = 1.24 × 10^−19^ J, and *φ*= 0.10, 0.20, 0.30, 0.40, *t* *_f_* is equal to 45.2 s, 7.6 s, 1.0 s, and 0.14 s, respectively.

The time required for an effective surfactant adsorption can be roughly estimated from Equation (34) assuming maximum surface excess: Γ(*t* = ∞) = 1/*A_s_* (*A_s_* = 50 Å^2^):

(57) $$t_{msa}\;\;=\;\;\pi /\left(4\;\;A_{s}^{2}\;C_{T}^{2}\;D_{s}\right)$$

Table 1 shows the values of *t_msa_* as a function of *D_app_* and *C_T_*. The value of *D_app_* depends on the molecular structure of the surfactant and other environmental parameters as the salt concentration, the temperature of the system, and the type of oil.

Comparison of the values of *t_f_* with the ones of *t_msa_* (last column of Table 1) shows that a typical non-ionic surfactant (*D_app_*= 10^−10^ m^2^/s) can stabilize emulsions with *φ* ≤ 0.30 if a minimum concentration of 5 × 10^−4^ M is used. The lower surfactant concentration (*C_T_* = 1 × 10^−4^ M) can only stabilize emulsions with *φ* ≤ 0.10. The exact value of the volume fraction in these estimations depends on the total interfacial area of the dispersion. The larger the interfacial area the higher the surfactant concentration required. For the extreme case of *D_app_* = 10^−12^ m^2^/s, a concentration of 5 × 10^−4^ M can only stabilize an emulsion with *φ* ≤ 0.10.

If the surfactant concentration is high *C_T_* = 5 × 10^−4^ M, and its diffusion constant fast *D_app_* = 10^−9^ m^2^/s, the initial drop size distribution of the emulsion is preserved [55]. In the opposite case, *D_app_* = 10^−12^ m^2^/s, *C_T_* = 1 × 10^−4^ M, complete destabilisation of the system is obtained. The most common cases happen between these extreme situations. They show an initial decrease in the number of drops due to the early destabilisation of the former DSD. Destabilisation occurs faster when the mean free path between the drops is small (high volume fraction, inhomogeneous spatial distribution of drops, etc.). As time passes, surfactant adsorption progressively increases until it reaches a point in which the repulsive potential generated by the surfactant is enough to preserve the current DSD. At this point the kinetic rates of coalescence and flocculation markedly change. The variation of number of aggregates as a function of time follows the prediction of Equation (52) for a completely different reason as the one discussed before. The repulsive force between the drops increases with time, not as a result of the surfactant redistribution among the drops, but due to the augment of the surfactant adsorption as a function of time. Notice also that in the case of non-deformable drops the diffusion constant decreases with *R_i_* and the repulsive forces augment. This might also cause a progressive stabilization of the system as a function of time.

In our calculations, *C_T_* = 5 × 10^−4^ M is the lowest surfactant concentration able to generate a substantial repulsive potential for *D_app_* ≥ 10^−10^ m^2^/s [55]. However, it must be kept in mind that in the referred simulations the total surfactant population is distributed among the surfaces of the drops. Hence, the mass balance does not include the surfactant solubility in the water phase. As a result it is expected that the surfactant concentration required for the attainment of the fastest stabilisation of the emulsion should be higher than 5 × 10^−4^ M.

As Equation (34) shows, a slow surfactant adsorption can be compensated with a high surfactant concentration. However, a low surfactant concentration can only be partially compensated with the increase of *D_app_*. That is the case of the systems with *C_T_* = 10^−4^ M and *φ* ≥ 0.20 [55]. A very low surfactant concentration does not generate a high repulsive force even if instantaneous adsorption occurs.

We also observed a marked effect of the hydrodynamic interaction in the most concentrated system (*φ* = 0.40). This system was expected to experience the highest destabilisation due to the short mean free path between the drops. However, this was partially the case, since the hydrodynamic interaction slowed down the flocculation of the drops considerably, producing a substantial lag time between the beginning of the simulation and the first coalesce event.

It should be remarked that the effect of the ionic strength was not considered in Ref. [55]. On the one hand, a high surface coverage generates a substantial repulsive force between the drops at low ionic strength (Figure 1). On the other hand, a high ionic strength (∼ 1 M NaCl) can screen completely the electrostatic interaction, eliminating any effect of dynamic adsorption on emulsion stability, for the case of an ionic surfactant. However, according to the results of Figure 9, an ionic strength as high as 0.55 M might not be not enough to eliminate the repulsive barrier between particles of nanometer size (σ*_T_* = 39 mCoul/m^2^ *, A_H_* = 4.27 × 10^−21^ J, *W* = 1.22).

Furthermore, it should be noticed that the value of the Hamaker constant used in the simulations of Ref. [55] was meant to represent the attraction between bitumen drops stabilized with a cationic surfactant [83]. A more typical value of *A_H_* for oils (∼10^−21^ J) should decrease the flocculation rate, diminishing the effect of the time dependent adsorption even more.

Taking into account the results of the simulations [55] and all the limitations outlined, the effect of dynamic adsorption is only expected to be relevant at low surfactant concentrations, *C_T_* ≤10^−4^ M, mostly between 0.20 ≤*φ*≤ 0.30. More precision requires further calculations employing the specific characteristics of the experimental system. These include not only the exact values of *φ, C_T_* *, and D_app_*, but also the initial DSD of the emulsion, the ionic strength of the aqueous solution, and the solubility of the surfactant (or its adsorption isotherm).

### 4.5. Ostwald Ripening

The Liftshitz-Slezov-Wagner (LSW) theory of Oswald ripening, predicts a linear variation of the cube of the average radius of a dispersion as a function of time ( $R_{c}^{3}$ vs. *t*) [84–85]. It also envisages a left-skewed drop-size distribution with a cut-off radius of 1.5 *R_c_*. These predictions are obtained assuming that:

- The particles are fixed in space.
- The system is infinitely dilute (implying the absence of interactions).
- The molecules of the internal phase are transported from one particle to another by molecular diffusion.
- The concentration of oil is the same through the whole system except in a direct neighbourhood of the particles, where it is given by the Kelvin equation.

LSW does not provide analytical expressions for the variation of *R_c_* during the transient period of ripening, nor does it consider the effects of flocculation and coalescence that simultaneously occur. At long times (in the asymptotic limit) LSW predicts a constant value for the Oswald ripening rate, V*_OR_*, equal to:

(59) $$V_{OR}\;\;=\;\;dR_{c}^{3}/dt\;\;=\;\;4\;\alpha \;D_{m}\;C\left(\infty \right)/9$$

Sakai *et al.* [86] studied the evolution of the DSD for several alkane/water systems in the absence of a surfactant. For a dilute dodecane in water emulsion (*φ* ≈ 2.29 × 10^−4^) a *V_OR_* rate equal to 4.0 × 10^−26^ m^3^/s was found. This rate is three times larger than the theoretical estimation (1.3 × 10^−26^ m^3^/s). Moreover, the DSDs of all measured systems were skewed to the right, in contradiction with the predictions of LSW. Using freeze-fracture electron microscopy, Sakai et al. [87] also reported direct observations of flocculation and coalescence of metastable benzene droplets under surfactant-free conditions. They observed small drops with diameters at 30–100 nm immediately after sonication, as well as aggregates of medium size (200–500 nm) composed of small droplets.

In Ref. [71] we revisited the evolution of a dodecane-in-water nanoemulsion in the absence of surfactant molecules. The research included simulations and experimental results. The main purpose of the investigation was to determine the effect of flocculation and coalescence in the temporal variation of $R_{c}^{3}$.

The first set of simulations followed the original algorithm of De Smet [67]. This code does not consider the movement of the drops. The initial DSD was Gaussian with *R_c_* (*t* = 0)= 30 nm. According to these calculations, *R_c_* does not increase prior to 800 s as a consequence of molecular diffusion. During this transient period the average radius of the emulsion slightly decreases due to the exchange of oil molecules between the drops. Moreover, *R_c_* does not increase until the first small particle dissolves. Hence, any increase of $R_{c}^{3}$ (*t*) prior to ∼ 800 s is the sole result of flocculation and coalescence (FC).

We carried out similar calculations with the ESS program including Ostwald Ripening (ESS+OR simulations). For these simulations 1000 non-deformable drops were used. The potential was completely attractive, characterized with a Hamaker constant, *A_H_* = 5.02 × 10^−21^ J. When the movement of the particles is incorporated, the simulations showed that *dR̄*^3^*/dt* changes linearly with time as a result of flocculation and coalescence: *dR̄^3^/dt*= *V_FC_* = 3.11 × 10^−22^ m^3^/s (r^2^ = 0.9979). This rate is four orders of magnitude higher than the theoretical value of *V_OR_* and cannot be attributed to Oswald ripening. Moreover, right-skewed distributions of particles were obtained [71].

This finding motivated the measurement of *R_c_* at short times. Dilute O/W dispersions of dodecane-in-water (*φ* = 2.3 × 10^−4^) were prepared using an aqueous solution of NaCl (0.5 M) to screen the electrostatic charge produced by the preferential adsorption of hydroxyl ions. Immediately after sonication, the light scattered by the emulsion was measured at 90° using a BI-200SM goniometer (Brookhaven Instruments). The mean diffusion coefficient was derived from the intensity autocorrelation function using a cumulant analysis [88].

**Figure 10.**
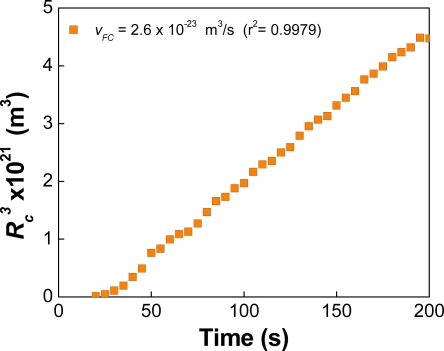
Experimental variation of the cube average radius as function of time. The data corresponds to a dodecane-in-water nanoemulsion produced by sonication.

Figure 10 shows the result of one of these measurements. A value of 2.6 × 10^−23^ m^3^/s was found for *dR̄*^3^*/dt.* This value differs in one order of magnitude from the one of the simulations, but is three orders of magnitude away from the theoretical value of *V_OR_* (1.3 × 10^−26^ m^3^/s). Moreover, if the emulsion is measured for a longer period of time (∼200 s), a small temporary plateau is reached, and the typical order of magnitude of OR (10^−26^ m^3^/s) is recovered.

The above result demonstrated that it is possible to obtain a linear relation of $R_{c}^{3}$ vs. *t* as a consequence of flocculation and coalescence, and not necessarily as a consequence of Ostwald ripening. It also suggests the existence of a small repulsive barrier that hinders flocculation and/or coalescence even at high ionic strengths.

In Section *3* we mentioned that in the absence of a strong repulsive force, the expression for the total number of aggregates of a dispersion (Equation (49)) is the same for suspensions and emulsions except for some minor restrictions regarding the counting of the aggregates. In the same reference [75] Danov et al. demonstrate that the formula for the concentration of *i*-particle aggregates is also equal to the one deduced by Smoluchowski *if the coalescence time is infinite*, or what is the same, if only flocculation occurs:

(60) $$n_{i}\;\;=\;\;n_{0}\;{\left(k_{f}^{slow}\;n_{0}\;t\right)}^{i-1}/{\left(1+k_{f}^{slow}\;n_{0}\;t\right)}^{i+1}$$

The average size of the particles in the system can be calculated substituting Equation (60) into:

(61) $$\bar{R}\;\;=\;\;\sum_{i\;=\;1}^{\infty }R_{i\;agg}n_{i}$$

Smoluchowski supposed that the dispersion was initially composed by particles of equal radius. Hence, some prescription was necessary to connect the size of the aggregates with the initial particle radius (*R*_0_). For example:

(62) $$R_{i\;agg}\;\;=\;\sqrt[{l}]{i}\;R_{0}$$

For coalescing emulsions *l* should be equal to 3. However, only certain values of *l* allow obtaining an analytical expression for *R̄*. A value of *l* = 1, consistent with the formation of linear aggregates, *R_i agg_* = *i R*_0_ produces a very simple formula:

(63) $$\bar{R}\;\;=\;\;R_{0}\;\left(1+k_{f}\;n_{0}\;t\right)$$

If the aggregates formed are not linear and/or coalescence occurs, Equation (63) is not valid and constitutes a very rough approximation to the mean particle radius. However, if we assume the validity of Equation (63) for a flocculating emulsion, a simple expression for *dR̄*^3^*/dt* results:

(64) $$V_{F}\;\;=\;\;d{\bar{R}}^{3}/dt\;\;=\;\;3\;R_{0}^{3}\;\left(k_{f}\;n_{0}\right){\left(1+k_{f}\;n_{0}\;t\right)}^{2}$$

where *V_F_* stands for the velocity of flocculation. The short-time limit of Equation (64) provides a very useful relation between *V_F_* and *k* *_f_* :

(65) $$\begin{array}{ccc} V_{F}\;\;=\;\;3R_{0}^{3}\;\left(k_{f}\;n_{0}\right) & \text{when}: & t\to 0 \end{array}$$

Notice that a real exponent *p* in Equation (63): *R̄* = *R*_0_ (1 + *k_f_* *n*_0_ *t*)*^p^*, still produces the same short-time limit, except for a constant multiplication factor *p.* Hence, use of the expression for the average radius found for Diffusion Limited Cluster Aggregation (DLCA): *R̄* =*R*_0_ (1 + *k_f_* *n*_0_ *t*)^1/^*^D_f_^* [89], also leads to Equation (65) with a different pre-factor: 3*/D_f_*. Moreover, the same limit is obtained for the case of Reactive Limited Cluster Aggregation (RLCA), where: *R̄*^3^ = $R_{0}^{3}$ exp (3 *k_f_* *n*_0_ *t/D_f_*) [90]:

(66) $$\begin{array}{ccc} d\;{\bar{R}}^{3}/d\;t\;\;=\;\;\left(3/D_{f}\right)k_{f}\;n_{0}\;R_{0}^{3} & \text{when}: & t\to 0 \end{array}$$

The data shown in Table 2 corresponds to the ESS + OR simulations referred above. As expected *V_F_* is proportional to *k_f_*. However, the quotient between *V_F_* and *k_f_* *n*_0_ $R_{0}^{3}$ does not appear to be related to any of the exponential factors suggested by Equations (65)–(66). Hence, it appears that the effect of the coalescence on the value of *dR̄*^3^*/dt* is significant. Yet, there appears to be a proportionality between the value of the flocculation rate and *dR̄*^3^*/dt.*

## 5. Modifications of ESS for the Calculation of Deformable Drops

The problem of drop deformation is formidably complex. Deformation results from the interplay between hydrodynamic and interaction forces. Hence it depends on all the parameters that affect these forces. As soon as deformation occurs, the geometrical part of the interaction potentials changes. Analytical forms for the potentials of truncated spheres are available in the literature, but they are function of the radius of the film between flocculated drops and its width. Moreover, the process of deformation itself involves several stages including the formation of a dimple, its evolution to a plane parallel film and the destabilisation of the film either by surface oscillation or by the formation of holes [91]. This complexity is additional to the one resulting from the simulation of all destabilisation processes that occur in emulsions. Hence, some drastic approximations are necessary. The methodology outlined in this section mostly corresponds to the thesis of Dr. Toro-Mendoza [92,93].

### 5.1. Regions of deformation

The present version of the ESS code has routines for non-deformable and deformable drops, but cannot simulate both types of drops during the same calculation. Thus, if the mode of deformable droplets is selected, it is assumed that the deformation of the drops occurs independently of the energy required for this process. Both types of simulation use the same equation of motion (Equation (12)) and follow the algorithm illustrated in Figure 2, but they differ in the way of calculating the diffusion constants, and the forces. Moreover, they differ in the criteria employed for the coalescence of drops.

In the mode of deformable droplets, the drops move as spheres until they reach the initial distance of deformation *h_0_*. When this occurs, a model of truncated spheres is used [6, 38, 94–95]. The transient time between the formation of the dimple and the surface oscillations that lead to the formation of a thin liquid film between flocculated drops is not taken into account. As soon as *r_ij_* < *R_i_* + *R_j_* + *h*_0_ the code calculates the dimensions of truncated spheres that are compatible with *h_0_* and *r_ij_*. Using the resulting film width and film radius, the program computes the potential of interaction between truncated spheres. In the absence of other coalescence mechanisms different from film drainage, the drops coalesce if the separation between their surfaces reaches a critical distance *h* = *h_crit_*.

In order to calculate the forces and diffusion constants three regions of approach are defined:

Region I: The distance of separation between the centres of mass of the drops *r_ij_*_,_ is greater than *R_i_* + *R_j_* + *h*_0_. In this case the drops maintain their spherical shape. Consequently:

(67) $$h=\;\;r_{ij}\;-R_{i}-R_{j}$$

(68) $$r_{film}=0$$

Region II: It covers the range of distances between the beginning of the deformation *r_film_* ≠ 0, and the attainment of the maximum film radius: *r_film_* = *r_f_*_max_ = $\sqrt{R_{i}\;h_{0}}$ (for *R_i_* < *R_j_*). Within these limits, the closest distance of separation between the surfaces of the drops is constant *h* = *h_0_* [6], and:

(69) $$h_{0}\;+\;\left(\sqrt{R_{i}^{2}-R_{i}\;h_{0}}+\sqrt{R_{j}^{2}-R_{i}\;h_{0}}\right)\;\;\;\;<\;\;r_{ij}\;\;\;\;<\;\;\;\;R_{i}+R_{j}+h_{0}$$

(70) $$r_{film}=\;\;\sqrt{R_{i}^{2}-{\left\{R_{i}\;\left(r_{ij}-h_{0}\right)/\left(R_{i}+R_{j}\right)\right\}}^{2}}$$

Region III: The film already attained its maximum radius, *r_film_* = *r_f_*_max_ = $\sqrt{R_{i}\;h_{0}}$, and it progressively drains until reaching a critical distance of approach. Within these separations:

(71) $$h_{crit}\;+\left(\sqrt{R_{i}^{2}-R_{i}h_{0}}+\sqrt{R_{j}^{2}-R_{i}h_{0}}\right)\;\;\;\;<\;\;\;\;r_{ij}\;\;\;<\;\;\;\;h_{0}+\left(\sqrt{R_{i}^{2}-R_{i}h_{0}}+\sqrt{R_{j}^{2}-R_{i}\;h_{0}}\right)$$

(72) $$h\;\;=\;\;r_{ij}+\left(\sqrt{R_{i}^{2}-r_{f\text{max}}^{2}}+\sqrt{R_{j}^{2}-r_{f\text{max}}^{2}}\right)$$

Notice that the drops behave as spheres in Region I (*r_ij_* > *R_i_* + *R_j_* + *h*_0_) even in the case in which the mode of deformable drops is selected. This means that the potential of interaction and diffusion constant in Region I correspond to spherical particles. When the distance of separation between the surfaces of the drops is less than *h_0_* (Regions II and III), the expressions of the potentials corresponding to truncated spheres are employed [38]. As explained in the next section, two additional potentials appear during the evolution of the film (Region II). They take into account: (a) the increase of interfacial area between a sphere and a truncated spheroid (extensional potential), and (b) the change of curvature of the interface (bending elasticity potential). These potentials contribute to the total potential of interaction and its force within Region II, but they assume a constant value in Region III and do not contribute to the value of the force in this zone.

### 5.2. Interaction forces between deformable droplets

As soon as deformation occurs, the analytical forms of the interaction potentials change. Analytical forms of the potentials for truncated spheres are available in the literature. However, they are expressed as a function of the radius of the film between flocculated drops and its width [6, 38, 95]. For example, the geometrical part of the van der Waals potential for truncated spheroids is equal to:

(73) VvdW  =  −AH12(2R2(l1−h)l1+(l2+h)+2R2(l1−h)h(l1+l2)+2ln(h(l1+l2)l1(l2+h))+rfilmh2−l1−hl2(2rfilm2hl1)−l1−R1−(l2−R2)2l1−2R1−h(2rfilm2hl1)−2(l2−R2)−h2l1−2R1−h(d−h2h) +2R2l22(l1−h)hl1(l1+l2)(l2+h)−2R22h(2l1−2R1−h)l12+rfilm2(l2+l2)(l2+l2−2R2) +2R22d(2l1−2R1−h)[(l2+h)(h+l2−2R2)−(l1−h)(l1−2R1−h)]+4R23(l1−h)(h+l2)(h+l2−2R2)−(l1−h)(l1−2R1−h)1(l1+l2)(l1+l2−2 R2)

where: *l*_1_=*h* + *R*_1_+ $\sqrt{R_{1}-r_{\text{film}}^{2}}$, *l*_2_=*h* + *R*_2_+ $\sqrt{R_{2}-r_{\text{film}}^{2}}$, *d* = $\sqrt{h^{2}+4r_{\text{film}}^{2}}$, *r_film_* is the radius of the thin liquid film between flocculated drops, and *h* is the closest separation between the surfaces of the drops.

Different types of interaction potentials for truncated spheres are found in the literature [38,95]. The researchers of the University of Sofia deduced most of them. They include van der Waals, electrostatic, oscillatory, depletion, steric, etc. These mathematical equations are expressed in terms of *h* and *r_film_*. Use of Equations (67)–(72), allows to recast those potentials in terms of *r_ij_*. Hence, it is possible to differentiate the potentials with respect to *r_ij_* using a package of symbolic algebra. In this way, the force is obtained for each deformation zone.

Additionally, two new contributions to the free energy appear. The first one is called dilatational or extensional energy and is caused by the increase of the interfacial area of the particle as it looses its spherical shape. The analytical form for the extensional potential corresponding to the change between a sphere and a truncated spheroid is [38, 94–95]:

(74) $$V_{d}\;\;=\;\;\gamma \;\pi \;r_{film,i}^{4}/\left(2\;R_{i}\right)$$

where *r_film_*_,_*_i_* is the radius of film formed by the truncated sphere *i*.

The variation of the interfacial curvature requires an additional amount of energy. This contribution can be positive or negative depending on the value of the spontaneous curvature of the interface:

(75) $$H_{0}\;\;=\;-1/R_{c,0}$$

where *R_c,0_* is the radius of curvature of the surface adopting its lowest free energy configuration. The energy of interfacial bending [38, 94–95] is equal to:

(76) $$V_{b}\;\;=\;\;-2\;\pi \;r_{film}^{2}\;B_{0}\;H$$

where *H* stands for the actual curvature of the interface:

(77) $$H\;=-1/R_{i}$$

and *B_0_* is the interfacial bending moment of a flat interface:

(78) $$B_{0}\;=\;\;-4\;k_{b}\;H_{0}$$

Constant *B_0_* is related to the bending moment in the theory of Helfrich [96]: *k_b_*. Theoretical estimations suggest a value of 5 × 10^−11^ N for *B_0_*. However, this value produces elastic potentials higher than 600 *k_B_T* for micron-size drops. In current calculations we adjust the value of *B_0_* in order to reproduce the expected bending energy of a 100 nm drop (31 *k_B_T*) [6]. This suggests a value of *B_0_* of 1.6 × 10^−12^ N.

**Figure 11.**
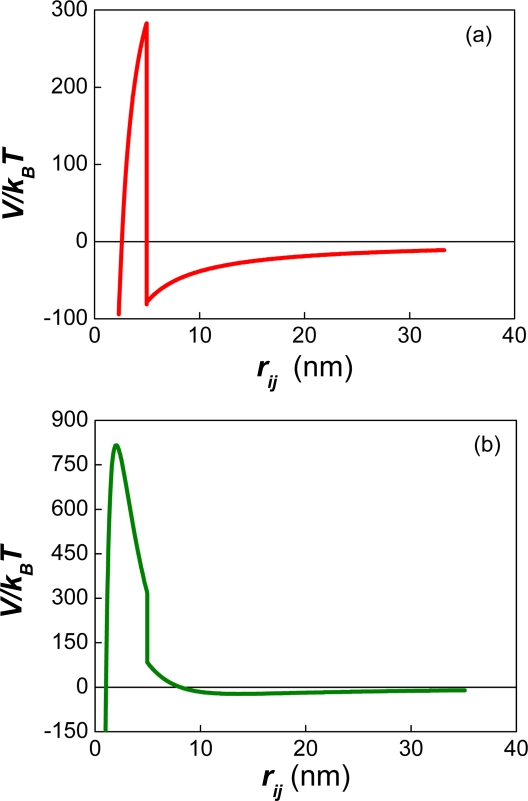
The curves correspond to the interaction potential between two deformable drops of dodecane suspended in water (*R_i_* = 3.9 μm, *A_H_* = 5.03 × 10^−21^ J, *C_s_* = 10^−4^ M, γ = 48.5 mN/m, *B_0_* = 1.6 × 10^−12^ N). (a) Sum of Extensional, Bending and van der Waals potentials. (b) Sum of Electrostatic and van der Waals potentials.

The potentials of surface deformation occur at close separation distances, within or nearby the same region of influence of the other potentials of interaction like electrostatic, steric, etc. In the absence of other repulsive potentials, deformation (dilatational + bending) generates two minima in the interaction potential separated by a repulsive barrier, similar to the ones exhibit by the DLVO potential (see Figure 1 and Figure 11). The presence of other repulsive contributions changes the smooth form of the total potential, increasing its value at short distances. This may also produce additional peaks and minima. Whatever the case, the passage of the particles over the closest repulsive barrier implies coalescence.

It is not yet possible to deduce a general analytical expression to relate *h*, *r_film_*, and *r_ij_* for all regions of deformation. Such an expression will round the sharp peaks of the total potentials shown in Figure 11, eliminating their present discontinuities. The acute changes of slope exhibited, result from the segmentation of the potential into three Regions (Equations (67)–(72)). However, all contributing potentials are differentiable within each domain and should not generate ill-define forces at the discontinuities.

It should be noticed, that Equations (74)–(78) rely on the interfacial tension, which in turn depends on the surface excess (Γ). Moreover, both free energies require the evaluation of the radius of the O/W/O film between flocculated drops (*r_film_*_,_*_i_*). This radius depends on the properties of the interfacial layer. Hence, in order to evaluate the forces in Equation (12) the number of surfactant molecules adsorbed to the interfaces of the drops has to be previously determined.

Accurate estimation of *h_0_* and *h_crit_* is *very difficult* since they result from a balance between hydrodynamic and interaction forces. On the one hand, a formal prescription for their evaluation is given in Ref. [97]. That type of calculations is too expensive in computational time, and cannot be incorporated into ESS. On the other hand, emulsions are polydisperse systems and the value of *r_film_* depends on the size of the flocculating drops and their interfacial tension. Figures 3 and 4 of Ref. [97] show the value of *h_0_* resulting from free energy calculations. We obtained an empirical form for *h_0_* in terms of the radius of *a drop* and its interfacial tension, *h_0_* (*R_i_*,γ), fitting the referred curves with an empirical polynomial:

(79) $$\begin{array}{l} h_{0}=\left(1.2932\;\;x\;10^{8}-8.6475\;x\;10^{-9}\;\text{exp}\left(-R_{i}/1.8222\;x\;10^{-6}\right)\right)\;\;x\\ \frac{\left(3.3253\;x\;10^{-9}+5.9804\;x\;10^{-9}\;\text{exp}\left(-\gamma /0.00402\right)\right)}{\left(3.3253\;x\;10^{-9}\;+\;\;5.9804\;x\;10^{-9}\;\text{exp}\left(-10^{-3}/0.00402\right)\right)} \end{array}$$

Equation (79) allows ascribing a value of *h_0_* **to each drop**. Since the value of *r_f_*_max_ cannot be larger than the radius of the smallest drop (*r_f_*_max_ = $\sqrt{R_{i}h_{0}}$), the value of *h_0_* selected for the flocculation of two drops of different sizes will be the one corresponding the smallest value of *r_f_*_max_.

In regard to *h_crit_*, the equation reported by Scheludko [98] and others [99–101] is used:

(80) $$h_{crit}\;\;=\;\;{\left(A_{H}\;\;\lambda_{crit}/128\;\gamma \right)}^{1/4}$$

where: λ*_crit_* ≈*r_film_*/10.

### 5.3. Calculation of the diffusion constant for deformable droplets

The form of the diffusion constant employed at every distance of separation corresponds to the shape of the approaching drops. Within Region I the same formalism used for non-deformable drops is employed [30]. For zones II and III several expressions are available in the program. They correspond to the rates of thinning *V_T_* of thin liquid films reported by Gurkov and Basheva [102]. These velocities depend on the force between the particles *F, h* and* r_film_*. Thus, they can be transformed into effective diffusion constants using the Einstein relation: *D* = *k_B_* *T/f*, where *f* is the friction coefficient *f* = *F/V_T_* (Equation (2)).

Most equations compiled by Gurkov and Basheva [102] require knowledge of the properties of the surfactant and/or the interfacial layer (Gibbs elasticity, viscosity, etc). Dr. Toro-Mendoza suggested Equation (81) based on a slight modification of the methodology previously used by our group for spherical drops [30].

(81) $$f_{corr}^{\left(2\right)}\;\;=\;\;\left\{\left(6\;u^{2}+\;\;4u\right)/\left(6\;u^{2}\;\;+\;13.0\;u\;+\;2\right)\right\}\left(4u^{3}\;\;R_{i}/r_{film}\right)$$

Thus, the analytical form of $f_{\text{corr}}^{\left(2\right)}$ proposed by Honig et al. [31] for spherical drops (expression in parenthesis in Equation (81)), used for *r_ij_* ≤ *r*_int_ is modified as soon as the drops change their spherical shape to form truncated spheres with a plane parallel film. Hence, the additional friction generated by the creation of two planar disks between flocculated drops, decreases the diffusion constant beyond the estimation of Honig et al.

### 5.4. Coalescence criteria for deformable drops

The process of coalescence of deformable droplets is involved. It is known that thin liquid films not necessarily drain until reaching *h_crit_* [7–10,103–105]:

(82) $$h_{rupture}\;\;\geq \;\;h_{crit}$$

Depending on the properties of its interfacial layers, films can collapse through the formation of holes [9–10] or due to the enhancement of surface oscillations [7–8, 105]. Stable Newton black films can also be formed, prolonging the stability of the flocs for months. In the case of small drops of nanometer size it seems unlikely that deformation takes place due to their high internal pressure.

By default, the mechanism of coalescence of deformable drops is the one of film drainage. As the drops approach, a plane parallel film forms and thins until *h_crit_* is reached. At this point, a new spherical drop is generated following Equation (17).

It is known that thin liquid films of macroscopic radii experience surface oscillations. In some circumstances the interfacial waves at each O/W interface may grow until they touch. In this case, a channel between the oil phases of the drops could form causing their coalescence. This mechanism is additional to the one of film drainage. Other possible process like the condensation of holes in common black films are also known to promote film rupture [9–10]. As in the case of surfactant adsorption, we use an algorithm which mimics the actual process. We do not attempt to simulate these processes in detail.

If the additional mechanism of surface oscillations is selected, a random number is assigned to the surface of each drop every time a pair of particles enters Regions II or III above. The random number varies between −1.0 and 1.0 units.

The amplitude (height) of each capillary wave (*λ*) at each interface is estimated as the product of the referred random number times the value of *h_crit_*.

(83) $$\lambda_{i}\left(t\right)=Ran\left(t\right)*h_{crit}$$

The time of existence of a doublet (*τ_ij_*) is estimated using one of two different procedures:

- *τ_ij_* is counted continuously from the moment a doublet enters Regions II or III until either *h* = *h_crit_* (coalescence occurs) or the double separates: *r_ij_* >*R_i_* + *R_j_* + *h*_0_. If the doublet separates *τ_ij_* is made equal to zero (its starting value at the beginning of the simulation).
- *τ_ij_* is estimated from the total number of time steps accumulated every time particles *i* and *j* enter zones II and III. In this case *τ_ij_* ≠ 0 after a couple of particles enter Regions II or III the first time.

At each time step, the value of *τ_ij_* is compared with a characteristic time deduced by Vrij [7] for the fastest increase in surface oscillations:

(84) $$\tau_{Vrij}\;\;=96\;\pi^{2}\;\;\gamma \;\eta \;h_{0}^{5}\;A_{H}^{-2}$$

Equation (84) was deduced assuming van der Waals interactions only. A more general expression requires knowledge of the second order differential of the free energy in terms *h* under certain restrictions. This differential changes abruptly for the case of deformable droplets. The program has the additional option to introduce the value of *τ_Vrij_* as input.

The value of the tension in Equation (84) is approximated by the average of the interfacial tension (*γ*) between the two flocculated drops (*γ_i_* + *γ_j_*)/2. *γ_i_* is calculated at each time step of the simulation from the number of surfactant molecules adsorbed using Equation (85):

(85) $$\gamma_{i}\;\;=\;\;\gamma_{0}\;\;+\;\;\left(\gamma_{cmc}-\gamma_{0}\right)\left(N_{s,i}/N_{s,i}^{\text{max}}\right)$$

Here, *γ*_0_, and *γ_cmc_* stand for the value of the O/W interfacial tension in the absence of surfactant molecules, and at the CMC of the surfactant employed. In the absence of stabilisers, *N_s,i_* = 0 at all times, and *γ_i_* = *γ*_0_.

Coalescence occurs whenever the total height of the surface oscillations is greater than *h_crit_*. The total height of the surface oscillations is approximated by:

(86) $$\lambda_{TOTAL}\;=\;\;\left(\lambda_{i}\;+\;\lambda_{j}\right)\left(\text{exp}\left(\tau_{ij}/\tau_{Vrij}\right)-1\right)$$

Equation (86) takes into account the fact that the surface oscillations increase exponentially with time [7–8,105].

It is clear from above, that all coalescence mechanisms are defined in terms of doublets. If aggregation of multiple particles occurs, Equation (86) is applied to each film formed between the flocculated particles.

The force routine is divided into two parts to account for non-deformable and deformable droplets. In the case of deformable droplets, the program estimates the value of *h_0_* for each drop according to Equation (79). Using this value and the distance of separation between the centres of mass (*r_ij_*), the region of approach can be determined (Regions I, II and III). The analytical expression for the force corresponding to the actual region of approach between each pair of drops is used.

### 5.5. Preliminary Results

The incorporation of deformable droplets is very recent [92–93]. We began the study of deformable droplets with the calculation of the coalescence time between two particles under the influence of van der Waals, extensional and bending potentials. All calculations began from a separation distance equal to *h* = *h_0_* (5 nm ≤*h*_0_ ≤15 nm). The particle radius was changed between 100 nm and 100 μm, for γ = 1 mN/m. Within these limits, the diffusion constant used (Equation (81)) markedly decreases as a function of the particles radius [93]. The coalescence times calculated for each particle radius correspond to the average value of 1000 simulations which only differ in the initial value of the random number generator. Only the mechanism of film drainage was considered for the coalescence of the drops.

For the 96-nm particles of Figure 9, the time for primary minimum flocculation changes between 6.35 × 10^−4^ s and 4.81 × 10^−3^ s (*A_H_* = 4.26 × 10^−21^ J, *h*(*t* = 0) = 20 nm). For deformable particles between 100 nm and 100 μm, the coalescence times are four orders of magnitude higher, increasing from a fraction of a second to 100 seconds (*A_H_* = 10^−20^ J, *h*(*t* = 0) = *h_0_*). These values are consistent with the times reported by Dickinson et al. for the coalescence time of emulsion drops with a planar oil-water interface (Figure 2 in Ref [106]).

The coalescence time between two *non-deformable* droplets increases steadily with the size of the particles. The coalescence time of *deformable* droplets increase between *R_i_* = 100 nm and *R_i_* = 5 μm (range 1), and between *R_i_* = 10 μm and *R_i_* = 100 μm (range 3). However, it decreases between 5 μm and 10 μm (range 2), generating a small peak in the curve of *t_c_* vs. *R_i_*. This particular behaviour is due to the opposite trends of interaction forces and hydrodynamic effects. Within range 1 the diffusion constant decreases abruptly causing an increase of the coalescence time as a function of the particle size. From 5 μm to 10 μm the diffusion coefficient also decreases but in a much lower rate. Hence the attractive force prevails decreasing *t_c_* as a function of *R_i_*. Within range 3, the interaction is still attractive. However, the values of *h_0_* and *r_f max_* = $\sqrt{R_{i}h_{0}}$, increase with the radius of the drop. The film drains at a slower rate, or what is the same, the effect of the diffusion constant prevails again, causing an increase in the value of *t_c_* as a function of *R_i_*.

In order to compare the effect of deformation on the rates of aggregation and coalescence, we evaluated an average (mixed) rate based on the variation of the number of *particles* in systems of 125 (*φ* = 0.10 and *φ* = 0.30):

(87) $$k_{FC}\;\;=\;\;1/t_{1/2}\;n_{0}$$

For *φ* = 0.10 the value of *k_FC_* for non-deformable droplets (3 × 10^−17^ m^3^/s) is two orders of magnitude higher than the one of deformable droplets (5 × 10^−19^ m^3^/s). For *φ*= 0.30 the difference reduces to only one order of magnitude (3 × 10^−16^ m^3^/s and 4 × 10^−17^ m^3^/s, respectively).

## 6. Conclusions

This review describes in detail the algorithm of Emulsion Stability Simulations developed by our group. It was not until very recently that we completed the routines for deformable droplets and Ostwald ripening. Consequently, it is now that we are ready to study the role of the surfactant molecule in systems exhibiting the concurrent occurrence of most destabilization processes. This is why we are confident that the most outstanding results from ESS are yet to be produced. However, we had shown that the previous studies related to the behaviour of non-deformable drops are very insightful. In particular they had remarked the role of the secondary minimum in the flocculation of drops, suggested a new threshold for the coalescence of drops, and demonstrated that the cube of the average radius of an emulsion can change linearly with time as a consequence of flocculation and coalescence.

## Figures and Tables

**Table 1.** Time required for maximum surfactant adsorption (*t_msa_*).

*D_app_* **(m^2^/s)**	*C_T_* **(M)**	*t_msa_* **(s)**
1 × 10^−9^	5 × 10^−4^	0.03
1 × 10^−9^	1 × 10^−4^	0.87
1 × 10^−10^	5 × 10^−4^	0.35
1 × 10^−10^	1 × 10^−4^	8.67
1 × 10^−12^	5 × 10^−4^	34.7
1 × 10^−12^	1 × 10^−4^	867

**Table 2.** Relationship between *k_f_* and V*_F_* (*R_0_* = 30 nm, *n_0_* = 2.01 × 10^18^ m^−3^).

**System**	***k_f_*** **(m^3^/s)**	***V_F_*** **(m^3^/s)**	**3** ***p***
**Dodecane/Water**	7.37 × 10^−18^	3.11 × 10^−22^	0.78
**Octane/Water**	6.62 × 10^−18^	2.58 × 10^−22^	0.72
